# Stage-specific transcription activator ESB1 regulates monoallelic antigen expression in *Trypanosoma brucei*

**DOI:** 10.1038/s41564-022-01175-z

**Published:** 2022-07-25

**Authors:** Lara López-Escobar, Benjamin Hänisch, Clare Halliday, Midori Ishii, Bungo Akiyoshi, Samuel Dean, Jack Daniel Sunter, Richard John Wheeler, Keith Gull

**Affiliations:** 1 Sir William Dunn School of Pathology, University of Oxford, Oxford, UK; 2 Department of Biochemistry, University of Oxford, Oxford, UK; 3 Division of Biomedical Sciences, Warwick Medical School, University of Warwick, Coventry, UK; 4 Department of Biological and Medical Sciences, Oxford Brookes University, Oxford, UK; 5 Peter Medawar Building for Pathogen Research, University of Oxford, Oxford, UK

**Keywords:** Parasite biology, Pathogens, Nuclear organization, Transcription

## Abstract

Variant surface glycoprotein (VSG) coats bloodstream form *Trypanosoma brucei* parasites, and monoallelic VSG expression underpins the antigenic variation necessary for pathogenicity. One of thousands of VSG genes is transcribed by RNA polymerase I in a singular nuclear structure called the expression site body (ESB), but how monoallelic VSG transcription is achieved remains unclear. Using a localization screen of 153 proteins we found one, ESB-specific protein 1 (ESB1), that localized only to the ESB and is expressed only in VSG-expressing life cycle stages. ESB1 associates with DNA near the active VSG promoter and is necessary for VSG expression, with overexpression activating inactive VSG promoters. Mechanistically, ESB1 is necessary for recruitment of a subset of ESB components, including RNA polymerase I, revealing that the ESB has separately assembled subdomains. Because many trypanosomatid parasites have divergent ESB1 orthologues yet do not undergo antigenic variation, ESB1 probably represents an important class of transcription regulators.

## Abstract (web summary)

The *Trypanosoma brucei* protein ESB1 is specific to the expression site body, where it activates pathogen monoallelic antigen transcription.

## Main

Monoallelic expression of a single gene family member underpins a molecular ‘arms race’ between many pathogens and their host, through host monoallelic immunoglobulin and pathogen monoallelic antigen expression. The unicellular parasite *Trypanosoma brucei* is an archetypal example, achieving antigenic variation through monoallelic expression of one of a library of thousands of variant surface glycoproteins (VSGs). VSG covers the entire cell surface in life cycle stages that inhabit the host bloodstream or are preadapted for transmission to the host^1^.

**Fig. 1:**
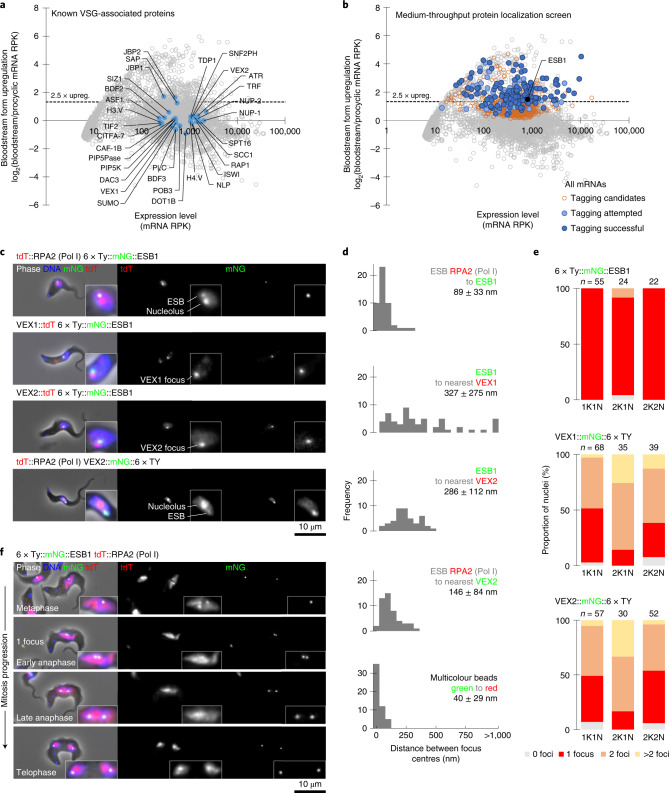
A protein localization screen identified ESB1. **a**,**b**, Degree of upregulation of *T. brucei* mRNAs in BSFs previously determined by RNA-seq^49^ highlighting known VSG monoallelic expression-associated factors (**a**) and candidates for tagging that we selected and successfully localized (**b**). **c**–**f**, Fluorescence microscopy analysis of ESB1 subcellular localization relative to known ESB-associated proteins. **c**, Representative images from at least *n* = 3 independent sample preparations of G1 (1K1N) cells from cell lines expressing one mNG-tagged and one tdT-tagged ESB-associated protein. For cells expressing VEX1 or VEX2, examples with one nuclear focus are shown. **d**, Histograms of pairwise distance measurements between the ESB1 focus, RPA2 ESB focus and the nearest VEX1 or VEX2 focus. For each, *n* ≥ 45 cells from one clonal cell line and all distances are significantly different (*P* < 10^−80^, two-tailed Mann–Whitney *U*-test). Multicolour beads are a control for measurement accuracy (true distance from zero). **e**, Number of mNG-tagged ESB1, VEX1 or VEX2 foci per nucleus in different cell cycle stages; *n* indicates the number of cells counted from one clonal cell line. The number of ESB1 foci significantly differs from that of VEX1 or VEX2 in 1K1N cell nuclei (*P* < 10^−9^, *χ*^2^-test). **f**, ESB1 localization in mitotic nuclei representative of *n* = 3 independent sample preparations. RPK, reads per kilobase. Source data

The single active VSG gene is transcribed by RNA polymerase I (Pol I)^2^ from a specialized bloodstream form (BSF) telomeric expression site (BES), where it is co-transcribed along with four or more expression site (ES)-associated genes (ESAGs) using a single promoter^3–5^. Switching of VSG is achieved by switching to transcription of one of several different telomeric BESs^4^ or replacement, by recombination, of the VSG in the active BES with one of the ~2,500 VSG gene and pseudogene variants elsewhere in the genome^6^. The active BES is found in a specialized Pol I-containing, non-nucleolar, nuclear structure called the expression site body (ESB)^2^, from which inactive BESs are excluded. The ESB is present only in BSF parasites^7^, despite procyclic forms (in tsetse fly) also employing Pol I-dependent transcription of their invariant surface coat (procyclin). Elegant biochemical candidate approaches and genetic screens of VSG expression have revealed the importance of epigenetic silencing^8^, telomere^9–12^ and chromatin factors^13–20^ and SUMOylation^21,22^. VEX proteins, required for exclusion of the inactive BESs^23,24^, associate the single active BES with the spliced leader array^25^ chromosomal locations. These contain the repetitive genes encoding a sequence which, after transcription and processing, is added to every trypanosome messenger RNA^26^. Hence, in addition to other properties, VEX proteins link an ESB-located exclusion phenomenon to an active VSG gene mRNA-processing capability. Notwithstanding these advances, bloodstream-specific factors (Fig. 1a and Extended Data Table 1) remain elusive and the statement that “No ESB-specific factor has yet been identified”^27^ still holds true. Here we used a medium-throughput localization screen to identify ESB-specific protein 1 (ESB1), which is expressed only in mammalian infectious forms and is localized specifically to the ESB. ESB1 is required for VSG expression and is located near the active VSG promoter, with overexpression activating inactive VSG promoters. We show that ESB1 is required for recruitment of some, but not all, ESB components, revealing that the ESB has separately assembled subdomains. Many trypanosomatid parasites have a divergent ESB1 orthologue, and therefore ESB1 potentially represents an important class of trypanosome transcription regulators.

## Results

**Extended Data Fig. 1:**
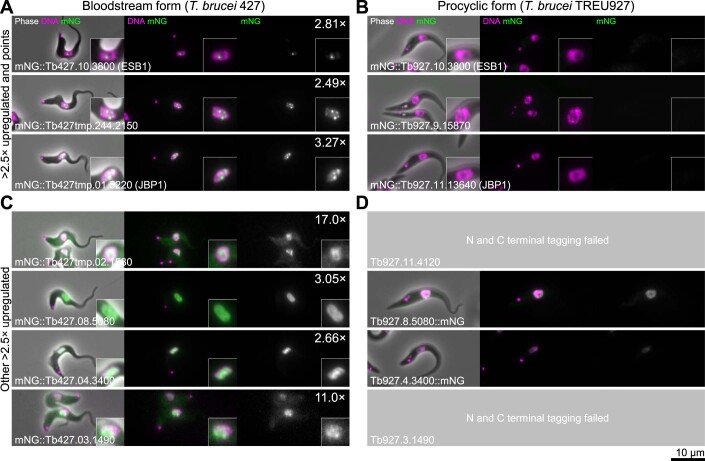
Bloodstream form-upregulated nuclear T. brucei proteins. Screening epifluorescence images of cell lines expressing tagged proteins, each image representative of *n* = 1 non-clonal cell line. Images for each cell line are laid in the same format: Left, an overlay of the phase contract (grey), mNG fluorescence (green) and Hoechst DNA stain (magenta). Middle, the DNA stain and the mNG fluorescence. Right, the mNG fluorescence in greyscale. Fold upregulation in bloodstream form cells is shown in the top right. **A)** Subcellular localisation of all 3 proteins >2.5× upregulated in bloodstream forms^49^ which localised to one or multiple points in the nucleus when tagged at the N terminus in bloodstream forms. **B)** Subcellular localisation of the 3 proteins in A) in equivalent procyclic form cell lines, shown at the same contrast levels. **C)** Subcellular localisation of the remaining 4 proteins >2.5× upregulated in bloodstream forms which localised to the wider nucleus when tagged at the N terminus in bloodstream forms. **D)** Subcellular localisation of the 4 proteins in C) in equivalent procyclic form cell lines, from TrypTag^28^, shown at the same contrast levels. We were unable to generate two cell lines.

**Extended Data Fig. 2:**
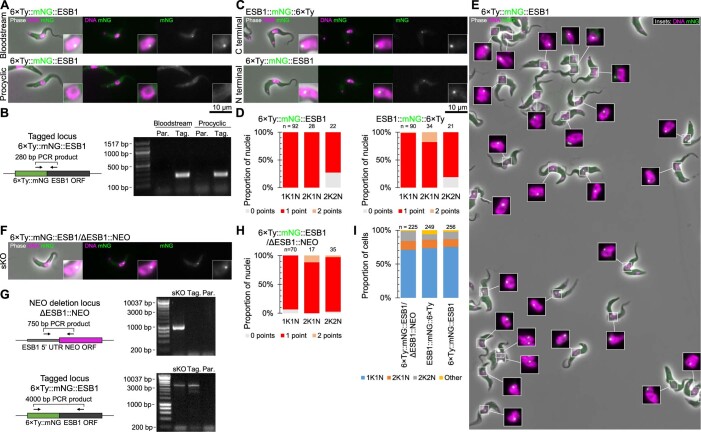
Tagging does not perturb ESB1 localisation or function. **A)** Clonal bloodstream form and procyclic form cell lines expressing Tb427.10.3800 or Tb927.10.3800 (ESB1) N terminally tagged with 6×Ty::mNG respectively were re-generated following the initial screen. Epifluorescence images representative of *n* = 1 clonal cell line of the localisation of the tagged protein by mNG fluorescence. **B)** Confirmation of the expected genetic modification of the cell lines in A) by PCR from genomic DNA using a forward mNG and a reverse ESB1 ORF primer. Schematic shows the primer binding sites, uncropped DNA gel shows the resulting PCR products from extracted genomic DNA from the tagged (Tag.) or parental (Par.) cell line. **C)** Epifluorescence images representative of *n* = 1 clonal cell line of bloodstream form cell lines expressing 6×Ty::mNG::ESB1 (N terminal tag), ESB1::mNG::6×TY (C terminal tag). **D)** Count of the number of points per nucleus at different stages of the cell cycle (1K1N, 2K1N and 2K2N) for N or C terminally tagged ESB1, from *n* = 1 replicate. **E)** Example field of view representative of *n* = 1 clonal cell line of cells expressing 6×Ty::mNG::ESB1 showing the strictly singular nature of the ESB1 nuclear focus. **F)** Epifluorescence image representative of *n* = 1 clonal cell line of a single knockout (sKO) bloodstream form cell line with one N terminally tagged ESB1 allele and the other deleted by replacement with a drug selectable marker. **G)** PCR validation of the sKO cell line. Schematics represent the deleted ESB1 ORF (top) and N terminally tagged locus (bottom) and primer binding sites, uncropped DNA gels shows the resulting PCR products from extracted genomic DNA. **H)** Count of the number of points per nucleus at different stages of the cell cycle for the sKO cell line, from *n* = 1 replicate. **I)** Proportion of cells at different stages of the cell cycle for the sKO in comparison to N or C terminal tagging, from *n* = 1 replicate, no changes *p* ≤ 0.05 from χ^2^ test. Source data

We performed a candidate protein localization screen of proteins of unknown function upregulated in the BSF^28^, and identified an ESB-specific protein. G1 BSF nuclei have one extranucleolar ESB^7,29^. From 207 candidates, 153 were successfully localized and only one (Fig. 1b), Tb427.10.3800, exhibited this localization (Fig. 1c and Extended Data Fig. 1a) whilst endogenous tagging in the procyclic form gave no detectable signal (Extended Data Figs. 1a and 2a,b). We named this protein ESB1.

We used well-characterized ESB markers to confirm ESB1 localization. Pol I is the founding component of the ESB and localizes to both the nucleolus and ESB in BSFs^7^. ESB1 lies extremely close to Pol I (RPA2) at the ESB (Fig. 1c), as confirmed by measurement of the distance between signal centre points (Fig. 1d). The ESB also has a VEX subcomplex involved in exclusion of inactive ESs^23^. ESB1 lies ~300 nm from the nearest VEX1 or VEX2 focus (Fig. 1c,d), significantly further than the distance from Pol I to VEX2 (Fig. 1d)^24^, suggesting that the centre of the Pol I body lies between ESB1 and VEX2. After S phase, cells still exhibit a single ESB before the nucleus undergoes closed mitosis. Unlike VEX1 and VEX2, ESB1 always localizes to a single focus per nucleus (Fig. 1e), whether tagged at the N or C terminus (Extended Data Fig. 2c–e). As *T. brucei* are diploid we also confirmed, by deletion of the untagged allele, that expression of N terminally tagged ESB1 in the absence of the wild-type allele gave the same localization (Extended Data Fig. 2f–h) and we saw no morphological or cell growth defect (Extended Data Fig. 2i). Similar to the ESB Pol I signal^29^, a second ESB1 focus formed only during anaphase (Fig. 1f). ESB1 is therefore specific to the ESB both spatially (localization) and temporally (life cycle stage-specific expression and cell cycle-dependent localization).

### ESB1 is necessary for active ES transcription

**Fig. 2:**
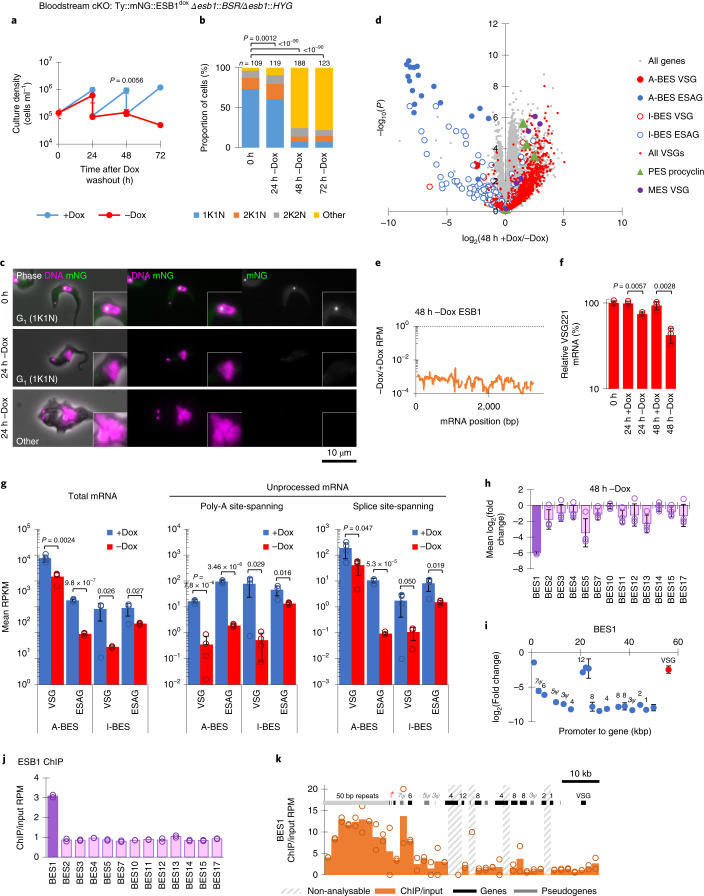
ESB1 is vital for BSFs and is required for transcription from the active VSG expression site. **a**–**f**, Cellular phenotype of BSF ESB1 cKO. Exogenous mNG-tagged ESB1 expression was maintained with 10 ng ml^–1^ doxycycline (+Dox) in the BSF ESB1 cKO (cell line validation shown in Extended Data Fig. 3a–d), and doxycycline washout (−Dox) induced the cKO phenotype. **a**, Culture growth (with subculture), mean ± s.d., *n* = 3 inductions. *P* value shown is at 48 h, two-tailed *t*-test, log cumulative growth. **b**, Counts of morphologically abnormal (‘Other’) cells following washout. *n* indicates number of cells counted, representative example from *n* = 3 inductions. *P* value derived from *χ*^2^-test. **c**, Representative images from *n* = 3 independent inductions showing mNG-tagged ESB1 signal before and after 24 h −Dox. **d**, Volcano plot of change in mRNA abundance as determined by RNA-seq after 48 h −Dox, *n* = 4 inductions (further time points shown in Extended Data Fig. 3e). *P* values derived from two-tailed *t*-test. A-BES and I-BES indicate active and inactive BES, respectively. **e**, Change in ESB1 ORF read coverage for 48 h −Dox, mean of *n* = 4 inductions. **f**, RT–qPCR quantitation of tA-ES VSG mRNA (VSG221) −Dox, mean ± s.d. from *n* = 3 inductions. *P* ≤ 0.05 derived from two-tailed *t*-test. **g**–**i**, Profile of transcript abundance change following ESB1 loss in BSFs. **g**, Changes in total and unprocessed mRNA grouped into A-BES or I-BES VSG(s) and ESAGs for cKO ±Dox. Mean ± s.d. from *n* = 4 inductions. *P* ≤ 0.05 derived from two-tailed *t*-test. Average change in transcript abundance averaged per BES (**h**) and per gene (**i**) for active BES plotted by distance from the promoter after 48 h −Dox; *n* = 4 inductions, mean ± s.d. **j**,**k**, ESB1 ChIP–seq shown as the ratio of ChIP to input DNA, plotting mean ratio per BES (**j**) and mean ratio in 2 kb bins across the active BES (**k**) (extended in Extended Data Fig. 4). Non-analysable bins had insufficient uniquely mapped reads from the input DNA. *n* = 2 replicates. Source data

**Extended Data Fig. 3:**
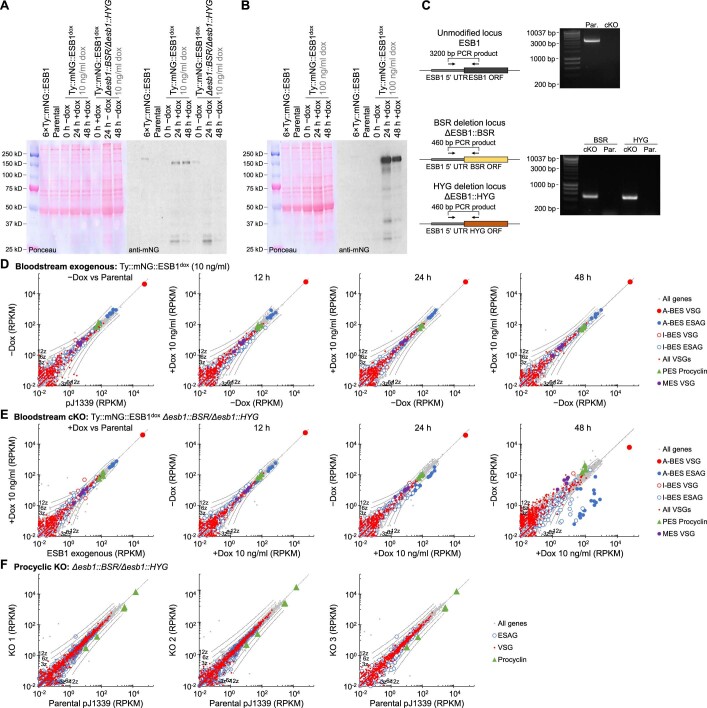
Generation and validation of an ESB1 conditional knockout. **A)** Western blot validation of the cKO cell line and the BSF pDex577 tagged ESB1 exogenous expression cell line (the intermediate in cKO generation), both induced with 10 ng/ml doxycycline. Predicted molecular weights for ESB1 are: 108 kDa (untagged), 137 kDa (Ty::mNG tag) and 145 kDa (6×Ty::mNG tag). The uncropped Ponceau-stained membrane and anti-mNG blot are shown. **B)** Western blot validation of the BSF pDex577 tagged ESB1 exogenous expression cell line induced with 100 ng/ml doxycycline for overexpression. **C)** Validation of genetic modifications of the ESB1 conditional knockout (cKO). Schematics represent the deleted and tagged loci and primer binding sites and orientations, uncropped DNA gels shows the resulting PCR products from extracted genomic DNA. **D)** mRNA abundance in the BSF pDex577 tagged ESB1 exogenous expression cell line, plotting RPKM of uniquely mapped RNAseq reads. The exogenous expression prior to addition of doxycycline (0 h) is plotted relative to the parental pJ1339 cell line. Other plots are 12, 24 and 48 h after addition of 10 ng/ml doxycycline relative to the cell line grown without doxycycline. Each shows *n* = 1 induction replicate. **E)** mRNA abundance in the cKO, plotting RPKM of uniquely mapped RNAseq reads. The cKO prior to doxycycline washout (0 h) is plotted relative to the BSF pDex577 tagged ESB1 exogenous expression cell line induced with 10 ng/ml doxycycline. Other plots are 12, 24 and 48 h after doxycycline washout relative to the cell line grown with 10 ng/ml doxycycline. Each shows *n* = 1 induction replicate. **F)** mRNA abundance in the procyclic form KO, plotting RPKM of uniquely mapped RNAseq reads for three clonal KO cell lines relative to the parental cell line.

To determine ESB1 function, we generated a BSF ESB1 conditional knockout (cKO) cell line (Extended Data Fig. 3a–e). ESB1 cKO gave undetectable levels of ESB1 protein by 24 h (Extended Data Fig. 3a), which caused a profound proliferation defect due to failure of cytokinesis and further rounds of organelle duplication (Fig. 2a–c). To detect any effect on BES transcription we used RNA sequencing (RNA-seq) to profile mRNA levels, which showed that ESB1 cKO caused a marked decrease (~250-fold) in ESAG mRNAs, predominantly those transcribed from the active BES (Fig. 2d and Extended Data Fig. 3e), associated with almost total loss of ESB1 transcript (Fig. 2e). mRNAs from the VSG gene in the active BES decreased ~eightfold (Fig. 2d), which we confirmed by quantitative PCR with reverse transcription (RT–qPCR) (Fig. 2f). The smaller decrease in VSG mRNAs is probably explained by the longer half-life of VSG mRNAs^30^.

To understand at what stage ESB1 functions in VSG and ESAG mRNA production, we analysed changes to nascent mRNAs in the BSF ESB1 cKO. Co-transcriptional trans-splicing and polyadenylation generate mature mRNAs^31^, enabling quantification of unprocessed transcript from RNA-seq reads spanning the spliced leader acceptor (SLAS) and polyadenylation (PAS) sites. Unprocessed ESAG and VSG mRNAs also dropped dramatically following ESB1 cKO (Fig. 2g), indicating that ESB1 cKO reduces active BES transcription rather than mRNA processing. Some low-processivity transcription of inactive ESs occurs^32,33^. ESB1 cKO caused a small reduction in unprocessed transcript from inactive ESs (Fig. 2g) and mRNAs transcribed from specific inactive ESs (Fig. 2h), while mRNAs from promoter-proximal ESAG genes in the active ES tended to be less strongly reduced (Fig. 2i). Therefore, the highly processive active ES transcription is ESB1 dependent, with ESB1 cKO leaving a little residual transcription such as seen at silent ESs.

**Extended Data Fig. 4:**
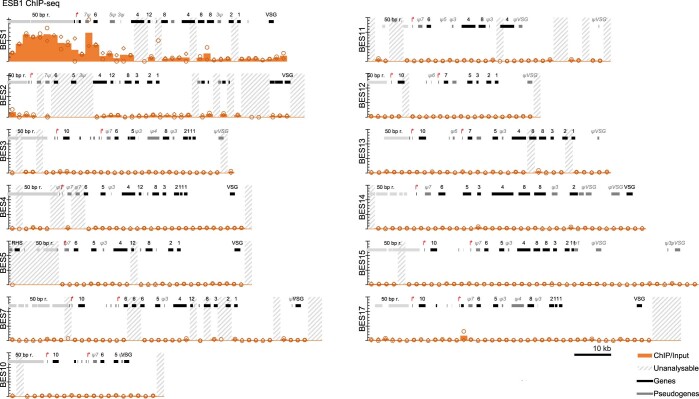
Extended presentation of ChIP data showing the active and inactive BESs. ESB1 ChIPseq in BSFs shown as the ratio of ChIP to input DNA in 2 kb bins across the active BES (BES1) and all inactive BESs. Unanalysable bins had insufficient uniquely-mapped reads from the input DNA. Extended version of Fig. 2K. *n* = 2 replicates, data points represent individual replicates and bars represent the mean.

A specific transcription activator would be predicted to associate only with the promoter region of the active ES. Therefore, we carried out ESB1 chromatin immunoprecipitation sequencing (ChIP–seq). Across the genome, the highest peak in ESB1 ChIP/input DNA ratio (30-fold background signal) was in the active ES. Among the ESs the active ES had the highest average ChIP ratio (Fig. 2j and Extended Data Fig. 4), due to a large peak between ~5 and 15 kb upstream and a smaller peak ~5 kb downstream of the Pol I promoter (Fig. 2k). The former corresponds to the imperfect 50 base pair (bp) repeats found upstream of all ESs^34,35^, but ESB1 associates with these repeats only at the active ES.

**Fig. 3:**
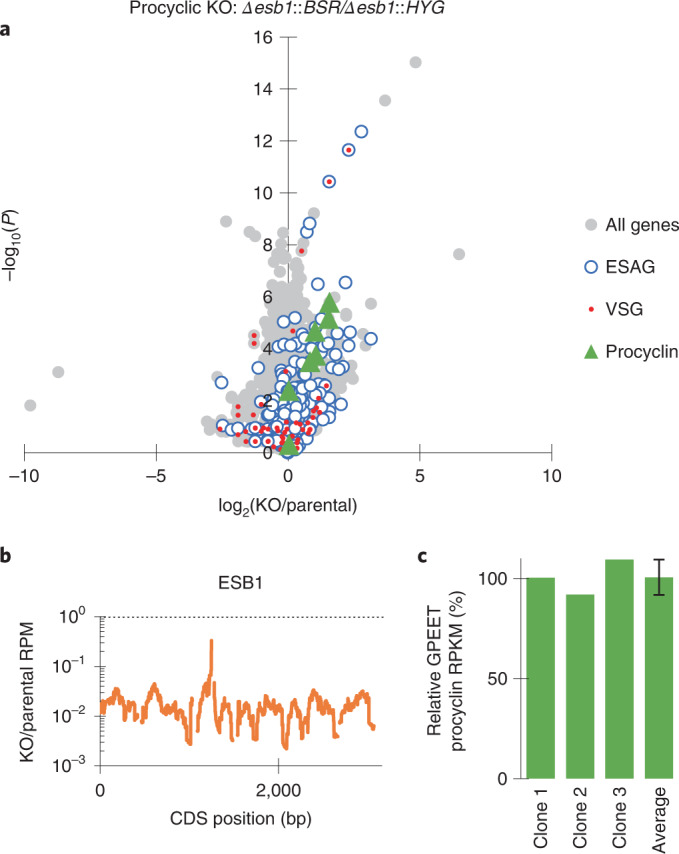
ESB1 is dispensable in procyclic forms. **a**–**c**, RNA abundance phenotype of procyclic form ESB1 KO. **a**, Volcano plot of change in mRNA abundance; *n* = 3 independent clonal cell lines (plotted individually in Extended Data Fig. 3f). *P* values derived from two-tailed *t*-test. **b**, Change in ESB1 ORF read coverage, mean of *n* = 3 clonal ESB1 KO cell lines. **c**, Abundance of GPEET procyclin in *n* = 3 clonal ESB1 KO cell lines relative to the parental cell line, as determined by RNA-seq. Fourth bar shows mean ± s.d.

Procyclic forms lack an active BES and an ESB and do not express ESB1, although they use Pol I for expression of their surface coat protein (procyclin) whose locus we refer to as a procyclin expression site. We tested ESB1 cryptic function in procyclic forms by deletion of both ESB1 alleles, which resulted in no apparent growth or morphology defect. RNA-seq confirmed normal high expression of GPEET procyclin and no major changes to other mRNA transcripts (Fig. 3 and Extended Data Fig. 3f). ESB1 is therefore vital in BSFs for monoallelic VSG expression, but is dispensable in procyclic forms.

**Extended Data Fig. 5:**
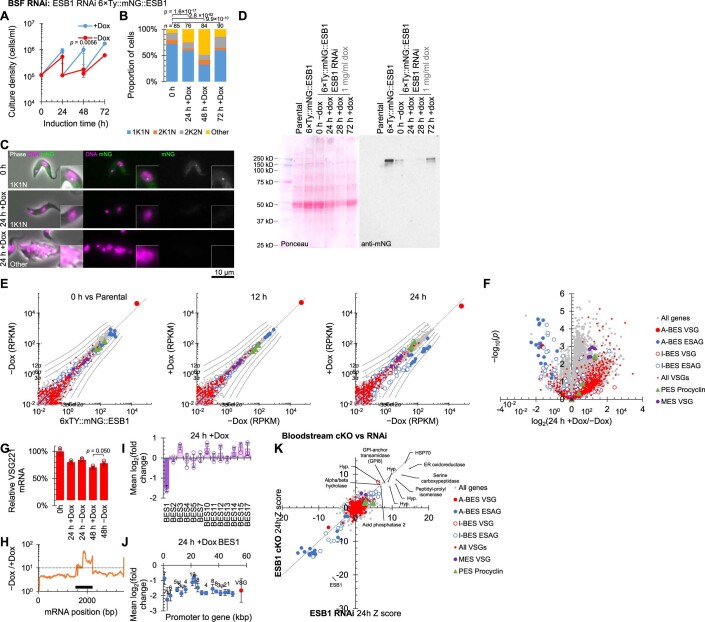
RNAi knockdown confirms ESB1 is vital and required for active BES transcription. Cellular phenotype of ESB1 RNAi knockdown. **A)** Growth curve of the ESB1 RNAi following doxycycline induction (+Dox) in comparison to uninduced (−Dox), using repeated subculture to maintain culture density under ~1×10^6^ cells/ml. Mean ± SD from *n* = 3 inductions. *p* shown at 48 h, two-tailed t test, log cumulative growth. **B)** Number of kinetoplasts (K) and nuclei (N) per cell, counted by light microscopy, at 24 h intervals following washout of doxycycline from the ESB1 cKO. representative example from *n* = 3 inductions. *p* from χ^2^ test. 1K1N, 2K1N and 2K2N are normal cell cycle stages. **C)** Representative images from *n* = 3 independent inductions of the ESB1 RNAi cell line before and after induction, showing a morphologically normal (1K1N) cell and a typical abnormal cell after 24 h induction. mNG signal is not detectable after 24 h induction. **D)** Uncropped anti-mNG Western blot validation of the ESB1 RNAi cell line. **E)** mRNA abundance in the BSF ESB1 RNAi cell line, plotting RPKM of uniquely mapped RNAseq reads. The uninduced cell line (0 h) is plotted relative to the parental 6×Ty∷mNG::ESB1 cell line. Other plots are 12 and 24 after addition of 1 mg/ml doxycycline relative to the cell line grown without doxycycline. Each represents *n* = 1 induction replicate. **F)** Volcano plot of change mRNA abundance determined by RNAseq 24 h +Dox, *n* = 4 inductions. *p* from two-tailed t test. A-BES and I-BES indicate active and inactive BES respectively. **G)** qRT-PCR measurement of active BES VSG mRNA relative to the parental cell line, mean ± SD from *n* = 3 inductions, *p* ≤ 0.05 shown from two-tailed t test. **H)** Change in RNAseq read coverage over the ESB1 open reading frame shows reduced ESB1 transcript, mean from *n* = 4 inductions. **I)** Average change in transcript abundance averaged per BES and **J)** per gene for the active BES plotted by distance from the promoter 48 h −Dox, *n* = 4 inductions, mean ± SD from *n* = 4 inductions. **K)** Correlation of per mRNA Z scores for the bloodstream ESB1 RNAi cell line with the ESB1 cKO after 24 h induction. Upregulated genes are labelled, and are ER stress associated. Source data

For further experiments, the BSF cKO phenotype was recapitulated with the more experimentally amenable RNA interference knockdown of ESB1 (Extended Data Fig. 5). mRNA abundance changes correlated extremely well with cKO (Fig. 2 and Extended Data Fig. 5k), with the same ESAGs and VSGs mRNAs reduced, as were the same set of 11 upregulated mRNAs (probably endoplasmic reticulum stress-associated; Extended Data Fig. 5k). The rapid lethality of the RNAi phenotype naturally led to the appearance of RNAi escape subpopulations;^36^ therefore, we analysed only early RNAi time points.

### ESB molecular composition depends on ESB1

**Fig. 4:**
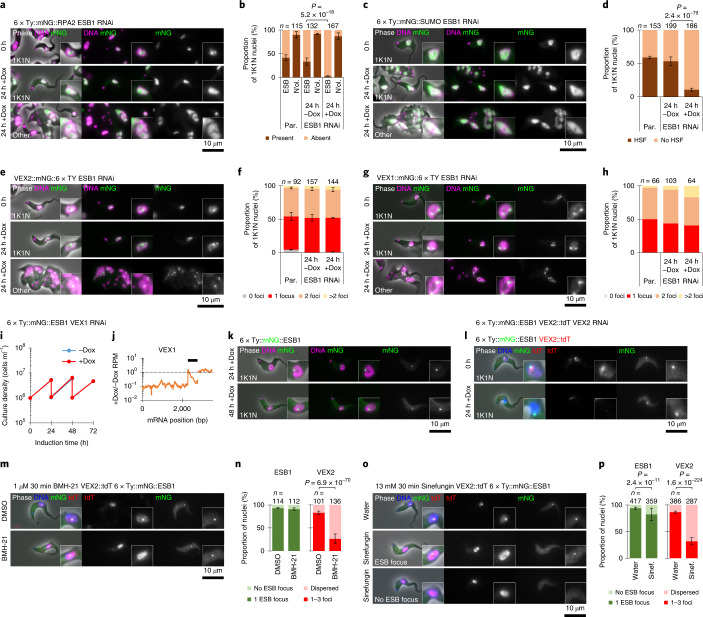
ESB1 is required for the formation of a subset of ESB substructures. **a**–**h**, Effect of doxycycline-inducible ESB1 RNAi knockdown (knockdown characterized in Extended Data Fig. 5) on mNG-tagged RPA2 (**a**,**b**), SUMO (**c**,**d**), VEX2 (**e**,**f**) and VEX1 localization (**g**,**h**). Each cell line was maintained without doxycycline (−Dox) then induced with 1 mg ml^–1^ doxycycline (+Dox). **a**,**c**,**e**,**g**, Representative fluorescence images from *n* = 3 (except for **g**, *n* = 1) independent inductions of tagged protein localization in morphologically normal and abnormal cells after 24 h RNAi induction. **b**,**d**,**f**,**h**, Counts of the number of cells with an RPA2-containing ESB focus and an RPA2-containing nucleolus (N’ol.) (**b**), with a HSF (**d**), the number of VEX2 foci (**f**) or the number of VEX1 foci in comparison with the parental (Par., no RNAi) cell line (**h**). Mean ± s.d. from three independent inductions (except for **h**, *n* = 1); *n* indicates total number of cells. *P* ≤ 0.05 derived from *χ*^2^-test. **i**–**k**, Effect of doxycycline-inducible VEX1 RNAi knockdown on mNG-tagged ESB1 localization. Culture growth (with subculture) (**i**) and change in VEX1 ORF read coverage showing effective knockdown determined by RNA-seq (**j**); *n* = 1 induction. **k**, Representative fluorescence images from *n* = 1 induction showing mNG-tagged ESB1 localization after VEX1 knockdown. **l**, Representative fluorescence images from *n* = 1 induction showing mNG-tagged ESB1 and tdT-tagged VEX2 localization after VEX2 knockdown. **m**–**p**, Effect of 1 μM BMH-21 (**m**,**n**) or 13 mM sinefungin (**o**,**p**) on ESB1 and VEX2 localization. **m**,**o**, Example fluorescence microscopy images following 30 min solvent control (DMSO or water) or compound treatment. **n**,**p**, Counts of the number of cells with focused or dispersed ESB1 and VEX2. Mean ± s.d. from three replicates; *n* indicates total number of cells, *P* ≤ 0.05 derived from *χ*^2^-test. Source data

We next determined whether ESB1, and thus active ES transcription, is required for the normal molecular composition of the ESB. We generated a panel of cell lines carrying the inducible ESB1 RNAi construct and tagged the following ESB-associated proteins: RPA2, SUMO (because the ESB is associated with a highly SUMOylated focus (HSF)^21^) and VEX1 or VEX2 (Fig. 4a–h). As shown by others, the ESB focus of RPA2 was visible in 40% of G1 nuclei (that is, when not occluded by nucleolar RPA2)^7^ and the HSF in ~60% of G1 nuclei^21^. After 24 h induction of ESB1 RNAi, RPA2 and SUMO were more dispersed through the nucleus and fewer nuclei had an ESB focus, in both morphologically normal and abnormal cells, while nucleolar RPA2 was unaffected (Fig. 4a–d). As seen previously, VEX1 and VEX2 localized to one or two foci in the nucleus. After 24 h induction of ESB1 RNAi, the localization pattern was unchanged, in both morphologically normal and abnormal cells (Fig. 4e–h). This indicates that ESB1 is necessary for both recruitment of Pol I to the ESB and higher local SUMOylation to form the HSF, but not for the formation of VEX foci.

**Extended Data Fig. 6:**
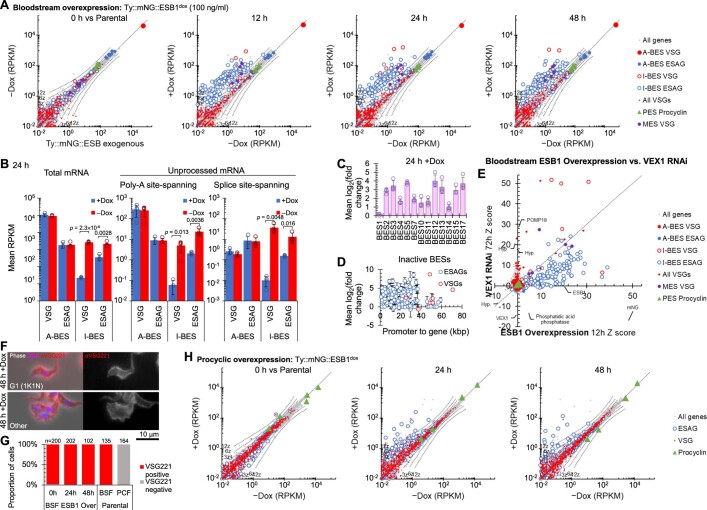
Extended analysis of the bloodstream and procyclic form overexpression analysis. **A)** mRNA abundance in the bloodstream form ESB1 overexpression cell line, plotting RPKM of uniquely mapped RNAseq reads. The overexpression prior to doxycycline addition (0 h) is plotted relative to the parental pJ1339 cell line. Other plots are 12, 24 and 48 h after 100 ng/ml doxycycline addition relative to the cell line grown without doxycycline. Each represents *n* = 1 induction replicate. **B)** Changes to total and unprocessed (not polyadenylated or not spliced) mRNA grouped into A-BES or I-BES VSG(s) and ESAGs for the overexpression + /−Dox. Mean ± SD from *n* = 4 inductions. *p* ≤ 0.05 shown from two-tailed t test. **C)** Average change in transcript abundance averaged per BES and **D)** per gene for the inactive BESs plotted by distance from the promoter 12 h +Dox, *n* = 4 inductions, mean ± SD. **E)** Correlation of per mRNA Z scores for the ESB1 overexpression, 12 h induction, with VEX1 RNAi, 72 h induction. Non-VSG and ESAG outliers are labelled. **F,G)** Anti-VSG221 immunofluorescence of the ESB1 overexpression line showing **F)** images of an example morphologically normal and abnormal cell after 48 h overexpression and **G)** counts of the proportion of VSG221-positive cells in comparison to the BSF and PCF cell lines. *n* numbers indicate number of cells counted from 1 induction replicate, no BSF changes *p* ≤ 0.05 from χ^2^ test. **H)** mRNA abundance in the procyclic form ESB1 overexpression cell line, plotting RPKM of uniquely mapped RNAseq reads. The overexpression prior to doxycycline addition (0 h) is plotted relative to the parental pJ1339 cell line. Other plots are 12, 24 and 48 h after 1 mg/ml doxycycline addition relative to the cell line grown without doxycycline. Each represents *n* = 1 induction replicate. Source data

The inverse, whether the ESB1 focus is VEX1 or VEX2 dependent, was analysed based on their depletion using RNAi and observing tagged ESB1 (Fig. 4i–l). VEX1 knockdown was confirmed using RNA-seq profiling of mRNA and, as previously described^23^, we saw derepression of inactive BESs (Extended Data Fig. 6e) with no growth defect (Fig. 4i,j). VEX2 knockdown was confirmed by carrying out knockdown in a cell line expressing tagged VEX2. ESB1 localization was unchanged following either VEX1 or VEX2 knockdown (Fig. 4k,l); therefore, formation of a singular ESB is not dependent on repression of inactive BESs by the VEX complex. The ESB1 and VEX2 compartments also have differing sensitivity to small molecule inhibitors. VEX2 foci became distributed following inhibition of Pol I transcription (BMH-21, an indirect Pol I inhibitor acting via DNA binding; Fig. 4m,n) or splicing (sinefungin; Fig. 4o,p), while the ESB1 focus was not strongly affected by either.

### ESB1 overexpression activates transcription from silent ESs

**Fig. 5:**
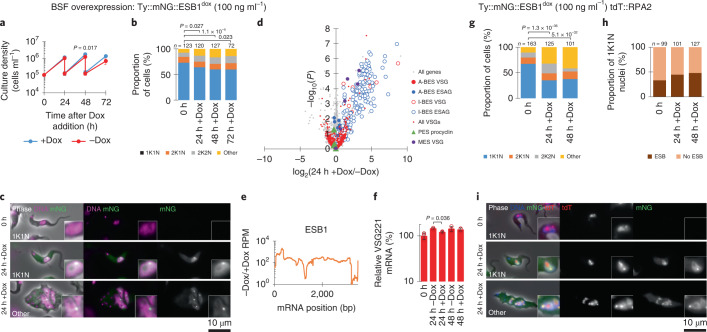
ESB1 overexpression in BSFs activates inactive BESs without affecting ESB formation. **a**–**f**, Cellular phenotype of mNG-tagged ESB1 overexpression in BSFs induced with +Dox. **a**, Culture growth (with subculture), mean ± s.d., *n* = 3 inductions. *P* value shown at 48 h, two-tailed *t*-test, log cumulative growth. **b**, Counts of morphologically abnormal (Other) cells following washout. *n* indicates number of cells counted, representative example from *n* = 3 inductions. *P* value derived from *χ*^2^-test. **c**, Representative images from *n* = 3 independent inductions showing mNG-tagged ESB1 signal before and after 24 h +Dox. **d**, Volcano plot of change in mRNA abundance as determined by RNA-seq for 24 h −Dox, *n* = 4 inductions (further time points shown in Extended Data Fig. 6a). *P* value derived from two-tailed *t*-test. **e**, Change in ESB1 ORF read coverage as determined by RNA-seq 24 h after washout, mean of *n* = 4 inductions. **f**, RT–qPCR quantitation of A-ES VSG mRNA (VSG221) +Dox, mean ± s.d. from *n* = 3 inductions. *P* ≤ 0.05 derived from two-tailed *t*-test. **g**–**i**, Effect of mNG-tagged ESB1 overexpression on tdT-tagged RPA2 localization. Counts of morphologically abnormal cells (**g**) and number of cells with an RPA2-containing ESB focus (**h**); *n* indicates number of cells counted from one induction, *P* ≤ 0.05 derived from *χ*^2^-test. **i**, Representative images from *n* = 1 replicate showing tagged protein localization before induction and in morphologically normal and abnormal cells after ESB1 overexpression. Source data

We then asked whether ESB1 overexpression could force ectopic BES expression and/or supernumerary ESB formation. Overexpression was achieved using a cell line with an additional inducible tagged ESB1 locus (using 100 ng ml^–1^ doxycycline; Fig. 5a–f and Extended Data Fig. 3b). In contrast to ESB1 cKO, overexpression yielded a small growth reduction and some cytokinesis defects (Fig. 5a–c). Overexpressed ESB1 still localized to the ESB, although with more dispersion in the nucleoplasm and cytoplasm, in both morphologically normal and abnormal cells (Fig. 5c). ESB1 overexpression in a cell line expressing tagged RPA2 showed that Pol I was not dispersed and was still localized at the single nucleolus and ESB (Fig. 5g–i), with an average separation of 76 ± 39 nm between the ESB1 focus and ESB. In BSFs, ESB1 overexpression therefore does not alter ESB number or form.

RNA-seq transcriptome profiling of the ESB1-overexpressing cell line showed a marked increase in mRNA levels, up to ~100-fold for VSGs and ESAGs transcribed from inactive BESs, while the mRNA levels of VSG and ESAGs transcribed from the active BES remained unchanged (Fig. 5d and Extended Data Fig. 6a, and confirmed using RT–qPCR in Fig. 5f) arising from a ~tenfold increase in ESB1 mRNA (Fig. 5e). mRNAs transcribed from specialized ESs containing metacyclic VSGs, normally expressed in the metacyclic life stage that is preadapted for transmission to the mammalian host, were similarly upregulated (Fig. 5d). Nascent inactive BES ESAG and VSG transcripts also markedly increased (Extended Data Fig. 6). mRNA transcribed from all inactive BESs increased (Extended Data Fig. 6c), with promoter-proximal ESAGs tending to be more strongly affected than promoter-distal ESAGs and VSGs (Extended Data Fig. 6d), unlike the phenotype of VEX1 knockdown (Extended Data Fig. 6e). ESB1 overexpression is therefore sufficient to cause activation of inactive BES transcription, although it may not be fully processive. All cells still expressed VSG221 (Extended Data Fig. 6f,g), therefore probably expressing multiple VSGs rather than switching to an alternative ES and VSG, whilst expression of procyclic form-specific surface proteins (procyclins) remained low (Extended Data Fig. 6a).

**Fig. 6:**
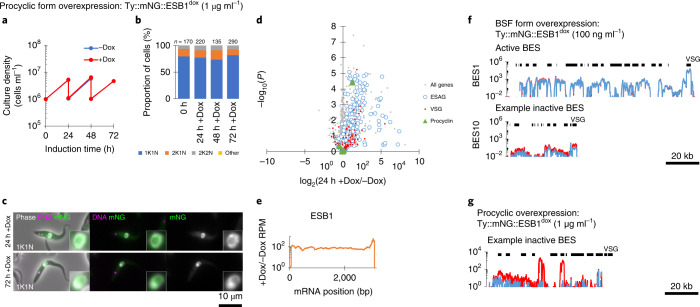
ESB1 overexpression in PCFs activates BES transcription without formation of an ESB. **a**–**e**, Cellular phenotype of mNG-tagged ESB1 overexpression in procyclic forms induced with 1 μg ml^–1^ doxycycline. Culture growth (with subculture, **a**) and counts of morphologically abnormal cells (Other, **b**) after induction; *n* indicates number of cells counted from one induction, no changes in *P* ≤ 0.05 from *χ*^2^-test. **c**, Example fluorescence images from *n* = 1 induction of overexpressed mNG-tagged ESB1 in procyclic forms. **d**, Volcano plot of change in mRNA abundance, *n* = 4 inductions (further time points shown in Extended Data Fig. 6h); *P* values derived from two-tailed *t*-test. **e**, Change in ESB1 ORF read coverage as determined by RNA-seq 24 h after induction, mean of *n* = 4 inductions. **f**,**g**, BES read coverage, as determined by RNA-seq 24 h after ESB1 overexpression, of the active and an example inactive BES in BSFs (**f**) (from Fig. 5), and an example inactive BES in procyclic forms (**g**); mean of *n* = 4 inductions. Further details given in Extended Data Fig. 8. Source data

Finally, we forced expression of tagged ESB1 in procyclic form cells (Fig. 6a–d and Extended Data Fig. 6h). Expression produced no growth or cytokinesis defects (Fig. 6a,b) and tagged ESB1 was nuclear, but did not localize to a single extranucleolar ESB-like focus (Fig. 6c). RNA-seq analysis showed a large increase (up to ~200-fold) in mRNA levels for ESAGs, consistent with the activation by ESB1 of transcription initiation at BES promoters normally inactive in the procyclic form (Fig. 6d). In this particular strain, we interrogated expression of the ESAGs and VSG from the sequenced BES^37^. Every ESAG transcribed from this BES was upregulated, typically ~three- to fivefold and up to ~80-fold (Fig. 6f,g). In contrast, VSG mRNAs (both published and from our de novo assembly of the transcriptome) were not strongly upregulated (Fig. 6d). We did not see a transcript from VSG 10.1, found in the sequenced BES, nor upregulation of any of the VSGs commonly expressed by this strain in BSFs during mouse infection^38^. This is despite ~50-fold overexpression of the ESB1 transcript relative to endogenous BSF expression (Figs. 5e and 6e). In regard to tagged ESB1 overexpression in the BSF, procyclin mRNA levels also remained unchanged (Fig. 6d). Hence ESB1 expression in procyclic forms activates BES transcription without formation of an ESB; however, transcription is either not fully processive to the most distal gene (VSG) or there is additional machinery required for VSG transcript maturation, processing and/or stability not expressed in the procyclic form—for example, CFB2 (ref. ^39^).

## Discussion

Antigenic variation in *T. brucei* relies on monoallelic expression of the VSG gene in the active BES. Our results provide the basis for a model whereby strong transcription activation of the active BES is counterbalanced by strong repression of all other BESs, and provides insights into the ESB subdomains that orchestrate these different functions.

**Extended Data Fig. 7:**
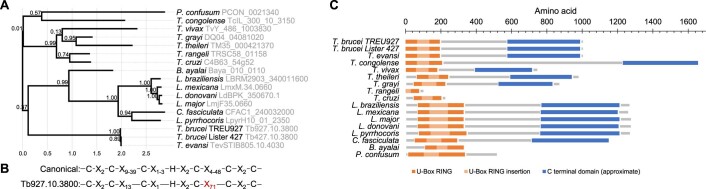
ESB orthologs among kinetoplastid parasites. **A)** Fast approximately maximum-likelihood phylogenetic tree of ESB1 and its orthologs. Node values are SH-like support. **B)** ESB1 U-box RING finger domain compared to the canonical sequence^74^ showing a large insertion. **C)** Domain structure of ESB1 and its orthologs. From sequence alone, the C terminal domain could not be detected outside of trypanosomatids.

We have identified ESB1 as both an ESB-specific protein and an ES transcription activator enriched near the Pol I promoter. We show that ESB1 is necessary for the high level of transcription from the active BES and that its overexpression activates only VSG-containing ESs and not procyclin loci. Importantly, both BESs and metacyclic VSGs are upregulated, and previous transcriptomics showed that metacyclic forms have upregulated ESB1 (ref. ^40^), indicating that VSG expression in the earliest VSG-expressing life cycle stage is ESB1 dependent. Ectopic expression of ESB1 in procyclic forms that never naturally express VSG was sufficient to activate BES promoter transcription, upregulating ESAGs located within a BES. However, ESB1 alone in procyclic forms was not sufficient for fully processive BES transcription and/or VSG mRNA processing. Interestingly, all trypanosomatid parasites, most of which do not undergo similar antigenic variation, have divergent ESB1 orthologues (Extended Data Fig. 7a). All orthologues have an N-terminal RING U-box domain while the weakly conserved C-terminal domain is not present in *Trypanosoma cruzi* and related *Trypanosoma* spp. and, when it is present, has very low sequence similarity to *T. brucei* (Extended Data Fig. 7b,c). This raises the prediction that Pol I transcription of protein-coding genes and their regulation may occur in other trypanosomatid parasites.

ESB1 alone was also not sufficient to support formation of the Pol I and ESB1 focus, because overexpression of ESB1 did not give rise to an ESB-like body/bodies in the procyclic form, or to supernumerary ESB-like bodies in the BSF. Moreover, multiple active BESs in multiple ESBs do not represent a stable state: in cells forced to express two VSGs from two BESs, both were recruited to a single ESB^41^. Given this, our ESB1 overexpression results suggest that the reasons for ESB absence (procyclic forms) or singularity (BSFs) are likely to be more complex than a threshold level of ESB1 protein. Phase separation, common in nuclear compartment formation, is among potential mechanisms for ESB formation where singularity could be achieved by emergent properties (Otswald ripening); however, ESB1 appears strongly chromatin associated, perhaps acting as a single nucleation site. These are open hypotheses for future work, and may also have important implications for understanding of switching between BESs.

Our work, taken with that of others, shows that the ESB is a complex nuclear body with multiple subdomains. The defining subdomain is a focus of Pol I around the active BES^7^, which also contains basal Pol I transcription factors^42^ and ESB1. This is associated with a HSF^21^. ESB1 is required for assembly of this subdomain. The BES is found in close proximity to one of the spliced leader array alleles^25^. Pol II transcription of this array generates the spliced leader RNA necessary for processing of all transcripts into mRNA. Each spliced leader array allele is found in a Pol II transcription focus^27^, and the proximity of one allelic copy to the ESB BES/Pol I subdomain provides a mechanism for efficient processing of the large amount of VSG mRNA. BES association with the ESB spliced leader array/Pol II subdomain requires VEX2 (ref. ^25^) and ESB BES/Pol I subdomain overlaps, or is adjacent to one VEX1 and VEX2 nuclear focus^23–25^. We show that assembly of these foci is separable, with assembly of the VEX foci not dependent on ESB1 and vice versa. Importantly, we show that the Pol I and ESB1 focus is strictly singular. This enhanced appreciation of the ESB in terms of spatially defined subdomains raises the possibility that it reflects an intrinsic functional architecture.

## Methods

### Parasite strains and cell culture

*Trypanosoma brucei* Lister 427 BSF was used because its expression sites are sequenced^43^ and assembled into contigs^8^. BSFs were grown in HMI-9 (ref. ^44^) at 37 °C with 5% CO_2_, maintained under ~2 × 10^6^ cells ml^–1^ by regular subculture. The active BES was BES1-containing VSG221 (also called VSG 427-2). *T. brucei* TREU927 procyclic form (PCF), selected because it is the original genome strain with genome-wide PCF localization data^28,45^, was grown in SDM-79 (ref. ^46^) at 28 °C and maintained between 6 × 10^5^ and 2 × 10^7^ cells ml^–1^ by regular subculture. We used PCF and BSF cell lines expressing T7 RNA polymerase, Tet repressor, Cas9 and PURO drug-selectable markers. These cell lines were generated using pJ1339, an expression construct that integrates into the tubulin locus^47^. To generate the Lister 427 BSF 1339 cell line, pJ1339 was linearized with HindIII and transfected into BSFs.

### Electroporation and drug selection

Linearized plasmid DNA or DNA (1–5 µg) from the necessary PCRs was purified by either phenol chloroform extraction (localization screen) or ethanol precipitation (other experiments), then mixed with 3 × 10^7^ cells (BSFs) or 1 × 10^7^ cells (PCFs) in 100 µl of Tb-BSF buffer^48^. Transfection was performed using the Amaxa Nucleofector IIb electroporator (program X-001, Lonza) in 2-mm-gap cuvettes. Cells were transferred to 10 ml of the appropriate prewarmed medium for 6 h, then the necessary drugs added to select for successful construct integration. Clonal cell lines were generated (except for the localization screen) by limiting dilution cloning. Cultures were maintained, with drug selection for any genetic modifications, using 0.2 µg ml^–1^ (BSF) or 1.0 µg ml^–1^ (PCF) Puromycin dihydrochloride, (2) 5.0 µg ml^–1^ (BSF) or 10 µg ml^–1^ (PCF) Blasticidin S hydrochloride, (3) 2.0 µg ml^–1^ (BSF) or 15 µg ml^–1^ (PCF) G-418 disulfate, (4) 5 µg ml^–1^ (BSF) or 25 µg ml^–1^ (PCF) Hygromycin B, 2.5 µg ml^–1^ (BSF) or (5) 5.0 µg ml^–1^ Phleomycin. Drug selection was removed for at least one subculture before an experiment.

### Medium-throughput BSF localization screen for ESB proteins

Tagging candidates were selected using published mRNA abundance data (RNA-seq)^49^, taking those with significantly upregulated transcripts (*P* < 0.05, two-tailed *t*-test) in BSFs relative to PCFs and prioritizing those >2.5-fold upregulated (Fig. 1b). Genes with unknown function were prioritized, with exclusion of VSG genes and pseudogenes, ESAGs, genes related to ESAGs and known invariant surface glycoproteins. Some known proteins—for example, ISG65 and GPI-PLC—were tagged as controls. We used other transcriptomic and ribosome footprinting datasets for further manual prioritization^49–53^. Tagging was at the N terminus unless the protein had a predicted signal peptide, in which case the C terminus was tagged. We attempted tagging of 207 proteins and successfully generated 153 tagged cell lines, seven with a nuclear signal (Extended Data Fig. 1).

### Endogenous tagging

To tag genes at the endogenous gene loci, we used long primer PCR and the pPOT plasmid series as the template to generate tagging constructs and, for BSF tagging, PCR to generate DNA-encoding single-guide RNA with a T7 promoter^54,55^. mNG^56^ was used for green fluorescent protein tagging, except for cell lines for ChIP where e-yellow fluorescent protein (eYFP) was used. pPOTv7-blast-mNG was used for the medium-throughput BSF localization screen. pPOTv6-blast-3Ty::mNG::3Ty was used for other experiments and, for simplicity, we refer to this as a 6×Ty::mNG tag. pPOTv7-hyg-tdTomato was used for tagging with a red fluorescent protein. PCR confirmed correct fusion of the mNG coding sequences (CDS) to the ESB1 CDS in PCFs.

### Exogenous expression and conditional knockout

For exogenous ESB1 (over)expression, the Tb927.10.3800 open reading frame (ORF) was amplified by PCR from TREU927 genomic DNA (gDNA) and cloned into pDex577 (ref. ^57^) and pDex777 (ref. ^58^) with a 1×Ty::mNG combined fluorescence reporter and epitope tag. These are doxycycline-inducible constructs, using a T7 promoter, that integrate into transcriptionally silent minichromosome repeats. pDex577/pDex777 constructs were linearized with NotI before transfection.

We titrated doxycycline concentrations to achieve a desirable exogenous Ty::mNG::ESB1 expression level by comparison with a cell line expressing 6×Ty::mNG::ESB1 from the endogenous locus using light microscopy, immunoblot (Extended Data Fig. 3a,b) and RNA-seq. We selected conditions to give (1) approximately endogenous expression level in BSFs (pDex577 with 10 ng ml^–1^ doxycycline), (2) overexpression sufficient to generate an aberrant BSF phenotype (pDex577 with 100 ng ml^–1^ doxycycline; Extended Data Fig. 3b) or (3) high overexpression in PCFs (pDex777 with 1 μg ml^–1^ doxycycline; Fig. 6e).

RNA-seq confirmed no major perturbation of cellular transcripts in BSFs expressing exogenous Ty::mNG::ESB1 from pDex577 with 10 ng ml^–1^ doxycycline (Extended Data Fig. 3d). We then deleted both endogenous ESB1 alleles (Extended Data Fig. 3c) while maintaining the cell line with 10 ng ml^–1^ doxycycline to generate the cKO cell line. For gene knockout we used long primer PCR to generate deletion and sgRNA constructs^54,55^ using pPOTv7 Hyg and pPOTv6 Blast. We confirmed knockout by PCR from genomic DNA, testing for loss of target gene CDS and their replacement by the drug selection marker. The cKO phenotype was observed by washing out doxycycline.

### PCR validation of endogenous locus ORF modification/loss

Key endogenous locus modifications were validated by PCR using template genomic DNA extracted using the DNeasy Blood & Tissue Kit (Qiagen). Primer pairs (Extended Data Table 2) spanned the endogenous DNA sequence to integrated DNA: for deletions, the gene 5′ untranslated region (UTR) to the drug selection marker ORF (Extended Data Figs. 2g and 3c) and, for tagging, the gene ORF to the fluorescent tag ORF (Extended Data Figs. 2b,g and 3c). PCR product size was checked by agarose gel electrophoresis (for primer sequences see Extended Data Table 2). In cases where both gene alleles were modified, the first allele modification was confirmed by PCR before the second allele was modified and confirmed.

### Inducible RNAi knockdown

For inducible ESB1, VEX1 or VEX2 RNAi knockdown we cloned a fragment (primer sequences shown in Extended Data Table 3) of the target gene ORF into a new doxycycline-inducible RNAi construct, pDRv0.5 (Supporting Information). This gives two copies of the fragment in reverse complement separated by a 150 nt stuffer. Two opposing T7 promoters under the control of doxycycline drive transcription of the resulting ‘stem-loop’. Cells were transfected with NotI linearized plasmid and selected using Hygromycin B. The construct integrates into the ribosomal RNA array. RNAi was induced using 1 µg ml^–1^ doxycycline.

To confirm effective knockdown, we introduced RNAi constructs into cell lines expressing an endogenously tagged copy of the target protein whose knockdown was confirmed by light microscopy (Extended Data Figs. 5c and 4l) and/or immunoblot (Extended Data Fig. 5d) and/or RNA-seq to determine the transcript abundance of the target gene (Fig. 4j).

For ESB1 RNAi knockdown in cell lines expressing endogenously tagged RPA2, SUMO, VEX1 or VEX2, we confirmed ESB1 knockdown by checking for the expected growth rate defect and change in proportion of cells at different cell cycle stages.

### Immunoblotting

Expression of endogenously tagged and exogenously (over)expressed proteins was confirmed by immunoblotting, using either 1:100 anti-mNG (mouse monoclonal IgG2c, ChromoTek 32f6, RRID: AB_2827566) or 1:100 anti-TY (from BB2 hybridoma, mouse monoclonal IgG1 (ref.^59^)) primary antibody and anti-mouse HRP-conjugated secondary antibody.

### Induction time series

RNAi and cKO cell lines were analysed as induction time series with paired induced and uninduced samples. Cells were subcultured to either 1 × 10^5^ cells ml^–1^ (BSFs) or 1 × 10^6^ cells ml^–1^ (PCFs), one sample without and one with the appropriate doxycycline concentration for induction. Each 24 h culture density was measured, samples taken and the remaining cells subcultured to either 1 × 10^5^ cells ml^–1^ (BSFs) or 1 × 10^6^ cells ml^–1^ (PCFs), with inclusion of doxycycline in the induced sample. For cultures with a strong growth defect, the culture was centrifuged at 1,200*g* for 5 min, the cell pellet resuspended in fresh medium and doxycycline added if needed, to maintain constant conditions. Growth defects were tested with two-tailed *t*-tests on log-transformed cumulative growth.

### Microscopy

Unless otherwise noted, light microscopy was carried out on live cells adhered to glass, with DNA stained by Hoechst 33342 (ref. ^60^), captured on a DM5500 B (Leica) wide-field epifluorescence microscope using a plan apo ×63/1.4 numerical aperture phase contrast oil-immersion objective (Leica, no. 15506351) and a Neo v.5.5 (Andor) sCMOS camera using Micro-Manager (v.1.4)^61^.

Kinetoplasts (K, mitochondrial DNA) and nuclei (N) in cells were counted from micrographs as a measure of cell cycle stage. K division normally precedes N division, giving 1K1N, 2K1N then 2K2N cells before cytokinesis. Cells with abnormal K/N numbers were classified as ‘Other’. Change in cell cycle stage distribution was tested with the *χ*^2^-test.

Spacing of point-like structures, one in green and one in red, was carried out by fitting a Gaussian in each channel then calculating centre point separation using ImageJ (v.1.50)^62,63^. Before analysis, chromatic aberration was corrected using reference images of 0.1 µm TetraSpeck multicolour fluorescent beads (ThermoFisher) adhered to glass^64^, and measurement error determined using green–red spacing in independent chromatic aberration-corrected images of multicolour fluorescent beads.

For blinded counts, one researcher identified and cropped in-focus nuclei of 1K1N cells from a mixture of test and control samples and saved each image with a randomized file name while generating an index. A second researcher classified the nuclei then unblinded using the index file.

For anti-VSG221 immunofluorescence, slides were prepared as for live-cell microscopy then cells were fixed with 2% formaldehyde for 5 min. Slides were then washed three times with PBS, incubated with 1:2,000 polyclonal rabbit anti-VSG^65^ for 1 h, washed three times with PBS, incubated anti-rabbit Alexa Fluor 647-conjugated secondary antibody for 1 h, washed three times with PBS and mounted with 50 mM phosphate-buffered 90% glycerol^60^.

### Transcriptomic analysis

RNA samples for each experiment were purified simultaneously by inducing separate samples at appropriately staggered intervals. A paired uninduced sample, maintained by the same pattern of subculture, was generated for each induction time point. From this time series, a time of primary interest was identified and three further paired samples were prepared. For each, 10^8^ cells were harvested by centrifugation at 3,200*g* for 90 s, the supernatant discarded and the pellet resuspended in 1 ml pf serum-free HMI-9. The suspension was centrifuged again at 10,000*g* for 30 s, the supernatant discarded by pipetting and the pellet flash-frozen in a dry ice/ethanol bath at −78 °C . Total processing time was <4 min. RNA was extracted using the RNeasy Mini Kit (Qiagen), eluted in 30 µl of nuclease-free water and stored at −80 °C. For RNA-seq, mRNA was enriched by polyA selection with complementary DNA generated by reverse transcription using a poly-dT primer, then subjected to 100 bp paired-end sequencing (BGISEQ-500) with a nominal insert size of 200 bp and >70,000,000 reads per sample.

To quantify transcript abundance from whole mRNAs rather than CDS, we first mapped the 5′ and 3′ UTRs using all our BSF Lister 427 RNA-seq data. SLASs and PASs were identified and assigned to protein-coding genes in the TriTrypDB^66,67^ release 45 of the *T. brucei* Lister 427 2018 genome using SLaPMapper^68^. SLASs and PASs observed once, used for <5% of transcripts from a gene or within a CDS were excluded from the analysis. No attempt was made to correct CDS based on SLASs/PASs. The most distant SLAS and PAS, within 5 kb of the CDS, defined the 5′ and 3′ UTR, respectively.

To quantify transcript abundance, fastq reads were mapped to the appropriate transcriptome using Burrows–Wheeler aligner-MEM (v.0.7.17) with default settings. Our Lister 427 2018 transcripts were used for 427 BSF samples and TriTrypDB^66,67^ release 45 for *T. brucei* TREU927 annotated transcripts for 927 PCF samples. An additional contig for the single sequenced and assembled *T. brucei* TREU927 BES^37^ was generated from NCBI GenBank nos. AC087700 (BES) and AF335471 (VSG), from which ESAG and VSG ORFs were identified and appended to the TREU927 transcriptome.

Because ESAGs have very similar sequences, transcript abundances were determined using uniquely mapped reads. Alignments were filtered to include only correctly mapped pairs with multiplexed analysis of projections by sequencing more than ten, excluding unmapped reads, secondary alignments and PCR or optical duplicates, using samtools view (v.1.7) with flags -q 10, -F 0×504 and -f 0×02. We confirmed that this accurately maps reads to the correct BES using simulated reads^69^. Using ART (v.2016-06-05)^70^, we generated an error model (using all RNA-seq data). Using this model and the measured insert size of 208 ± 78 bp, we simulated data with 500-fold coverage of Lister 427 2018 transcripts and aligned them to the Lister 427 genome. Without filtering, between 54.9% (BES5) and 90.2% (BES10) of simulated reads were mapped to the correct BES; with filtering, this improved to >99.75% for all BESs.

Reads per kilobase per million (RPKM) was calculated from samtools idxstats. Mean read coverage was calculated from samtools depth, with flags -aa -d 10000000, then converted to counts per million reads. For time points with a single replicate, z-intervals were calculated from variation between uninduced samples (*n* = 3). Standard deviation of log fold change for transcripts binned by RPKM (20 bins, *n* > 70 genes per bin) was calculated and fitted to a third-order polynomial for plotting. For time points with multiple replicates, mean and two-tailed *t*-test *P* values of log_2_ fold change were calculated for volcano plots.

Immature/nascent transcripts were quantified by filtering the alignments for reads spanning a SLAS or PAS, indicating that trans-splicing or polyadenylation, respectively, may not yet have occurred. Reads were scored by the sum frequency of use of spanned sites (1.00 if the only site, 0.05 if spanning a site used 5% of the time, 0.97 if spanning two sites used 63 and 34% of the time, respectively, and so on) then normalized to a score per 1,000,000 reads (that is, reads per million like).

Active BES VSG (VSG221) RT–qPCR) used a one-step protocol from total RNA, with β-tubulin as a control (primer sequences shown in Extended Data Table 4). Total RNA was diluted to 500 ng µl^–1^ based on OD260, and RT–qPCR performed using the QuantiTect SYBR Green RT-PCR Kit (Qiagen, no. 204243) with the manufacturer’s recommended reaction composition and thermocycle on a Mx3000P QPCR machine (Agilent). Specific PCR product was confirmed by gel electrophoresis and product melt curve analysis, with no template or primer controls. A six-step, threefold dilution series from 1:3^0^ (1:1) to 1:3^6^ (1:279) of parental cell line RNA was used to confirm VSG and tubulin critical cycles falling within the linear range. Mean VSG221 to tubulin critical cycle was determined in triplicate using 1:10 diluted RNA samples and MxPro QPCR Software (Agilent).

For de novo transcriptome assembly we used Trinity (v.2.11.0) guided by Harvard FAS best practices^71^. Sequencing errors were first corrected using Rcorrector (v.1.0.4)^72^ and uncorrectable reads were removed, then any remaining adaptors and low-quality sections were trimmed with Trim Galore! (v.0.6.0) with flags length 36, -q 5,–stringency 1 and -e 0.1. Finally, read ends that exactly matched four or more bases of the 3′ end of the *T. brucei* spliced leader sequence were trimmed. Trinity, using default settings, generated the assembly.

### ChIP–seq

For ChIP–seq we used the following optimized protocol^73^: 5 × 10^8^ BSF-expressing YFP::ESB1 at 1 × 106 cells ml^–1^ were fixed with a 1/8 volume of formaldehyde (50 mM HEPES-KOH pH 7.5, 100 mM NaCl, 1 mM EDTA, 0.5 mM EGTA and 8% formaldehyde) for 20 min at room temperature, followed by the addition of a 1/13 volume of 2 M glycine and kept on ice. Fixed cells were rinsed with 35 ml of PBS, resuspended in 35 ml of lysis buffer 1 (50 mM HEPES-KOH pH 7.5, 140 mM NaCl, 1 mM EDTA, 10% glycerol, 0.5% NP-40, 0.25% Triton X-100 and protease inhibitors) and centrifuged at 4,000*g* for 15 min. The pellet was resuspended in 35 ml of lysis buffer 2 (10 mM Tris-HCl pH 8.0, 200 mM NaCl, 1 mM EDTA, 0.5 mM EGTA and protease inhibitors) and centrifuged at 4,000*g* for 15 min. The pellet was resuspended in 4 ml of lysis buffer 3 (10 mM Tris-HCl pH 8.0, 100 mM NaCl, 1 mM EDTA, 0.5 mM EGTA, 0.1% Na-deoxycholate, 0.5% N-lauroylsarcosine and protease inhibitors) and sonicated (27 s on/30 s off, eight cycles) using a VCX 130 PB (Sonics & Materials). A 1/10 volume of 10% Triton X-100 was added to the sonicated lysate and centrifuged at 21,000*g* for 10 min to pellet debris, and the supernatant collected. YFP-tagged proteins were immunoprecipitated with rabbit anti-green fluorescent protein (Invitrogen, no. A11122, RRID: AB_221569) preconjugated with Protein-A magnetic beads (Dynal). Beads were washed with 1 ml of RIPA buffer (50 mM HEPES-KOH pH 7.5, 500 mM LiCl, 1 mM EDTA, 1.0% NP-40 and 0.7% Na-deoxycholate) seven times and rinsed with with 50 mM Tris-HCl pH 8.0, 10 mM EDTA and 50 mM NaCl. DNA was eluted with 200 µl of elution buffer (50 mM Tris-HCl pH 8.0, 10 mM EDTA and 1.0% SDS) at 65 °C for 30 min. Crosslinking was reversed by incubation at 65 °C overnight. The sample was treated with RNase A (0.4 mg ml^–1^, QIAGEN) at 37 °C for 2 h and Proteinase K (0.420 mg ml^–1^, Life technologies) at 55 °C for 2 h, then purified using a PCR purification kit (QIAGEN).

Both input and ChIP DNA were sequenced by 50 bp single-end sequencing (DNBSEQ). The ChIP/input ratio was calculated from reads uniquely mapped to the 2018 resequence of *T. brucei* Lister 427, using Burrows–Wheeler aligner and samtools. Only reads with multiplexed analysis of projections by sequencing greater than three were included and unmapped reads, secondary alignments and read PCR or optical duplicates were excluded, using samtools with flags -q 3 and -F 0×504. Mean ChIP/input ratio was calculated for each BES contig and calculated genome wide in 2 kb bins. Bins with fewer than four uniquely mapped input DNA reads were classed as non-analysable, due to either insufficient input DNA or an insufficiently unique sequence for mapping of 50 bp reads.

### Reporting summary

Further information on research design is available in the Nature Research Reporting Summary linked to this article.

## Supplementary information

Supplementary Information Supplementary Tables 1–4.

Reporting Summary

Supplementary Data 1 pDR0.5 RNAi plasmid map.

## Figures and Tables

**Extended Data Fig. 8:**
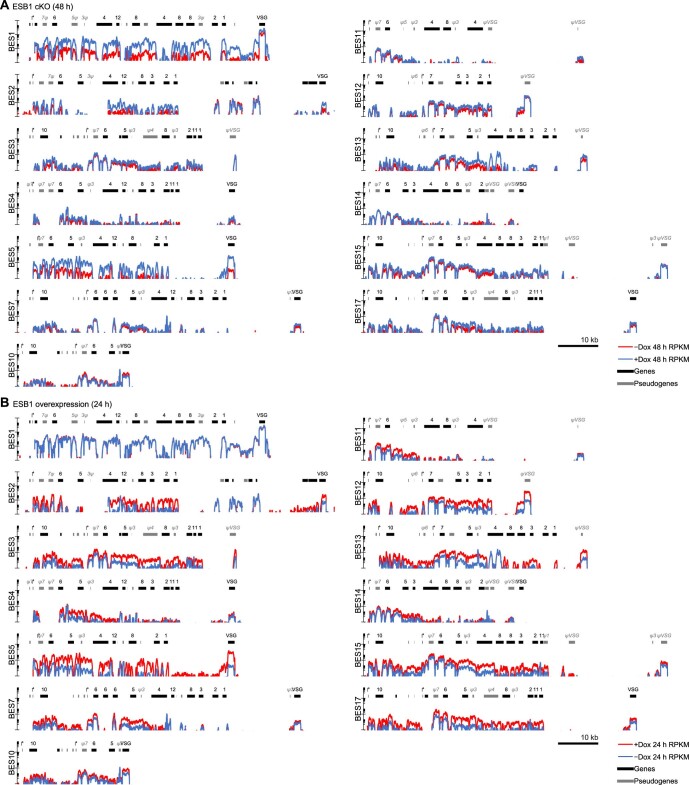
Extended presentation of RNAseq data showing the active and inactive BESs. **A,B)** Read coverage of the active BES (BES1) in comparison to all sequenced inactive BESs, determined by RNAseq, for **A)** the BSF ESB1 cKO (characterised in Fig. 2A-F) 48 h after induction and **B)** the BSF ESB1 overexpression (characterised in Fig. 5A-F) 12 h after induction. The latter is an extended version of Fig. 6F. Mean of *n* = 4 inductions.

## Data Availability

RNA-seq and ChIP–seq data are available via the NCBI sequencing read archive under BioProject accession no. PRJNA784098. Source data are provided with this paper.

## Source data

Source Data Fig. 1 Statistical source data.
Source Data Fig. 2 Statistical source data.
Source Data Fig. 4 Statistical source data.
Source Data Fig. 5 Statistical source data.
Source Data Fig. 6 Statistical source data.
Source Data Extended Data Fig. 2 Statistical source data.
Source Data Extended Data Fig. 5 Statistical source data.
Source Data Extended Data Fig. 6 Statistical source data.
