# Stage-specific transcription activator ESB1 regulates monoallelic antigen expression in *Trypanosoma brucei*

**DOI:** 10.1038/s41564-022-01175-z

**Published:** 2022-07-25

**Authors:** Lara López-Escobar, Benjamin Hänisch, Clare Halliday, Midori Ishii, Bungo Akiyoshi, Samuel Dean, Jack Daniel Sunter, Richard John Wheeler, Keith Gull

**Affiliations:** 1 Sir William Dunn School of Pathology, University of Oxford, OX1 3RE; 2 Department of Biochemistry, University of Oxford, OX1 3QU; 3 Division of Biomedical Sciences, Warwick Medical School, University of Warwick, Coventry CV4 7AL; 4 Department of Biological and Medical Sciences, Oxford Brookes University, OX3 OBP; 5 Peter Medawar Building for Pathogen Research, University of Oxford, OX1 3SY

## Abstract

Variant surface glycoprotein (VSG) coats bloodstream form *Trypanosoma brucei* parasites and monoallelic VSG expression underpins the antigenic variation necessary for pathogenicity. One of thousands of VSG genes is transcribed by RNA polymerase I (Pol I) in a singular nuclear structure called the expression site body (ESB) but how monoallelic VSG transcription is achieved remains unclear. Using a localisation screen of 153 proteins, we found one, ESB-specific protein 1 (ESB1), which localised only to the ESB and is expressed only in VSG-expressing life cycle stages. ESB1 associates with DNA near the active VSG promoter and is necessary for VSG expression, with overexpression activating inactive VSG promoters. Mechanistically, ESB1 is necessary for recruitment of a subset of ESB components, including Pol I, revealing the ESB has separately assembled sub-domains. As many trypanosomatid parasites have divergent ESB1 orthologs yet do not undergo antigenic variation, ESB1 likely represents an important class of transcription regulators.

Monoallelic expression of a single gene family member underpins a molecular “arms race” between many pathogens and their host, through host monoallelic immunoglobulin and pathogen monoallelic antigen expression. The unicellular parasite *Trypanosoma brucei* is an archetypal example, achieving antigenic variation through monoallelic expression of one of a library of thousands of variant surface glycoproteins (VSGs). VSG covers the entire cell surface in life cycle stages which inhabit the host bloodstream or are pre-adapted for transmission to the host^1^.

The single active VSG gene is transcribed by RNA polymerase I (Pol I)^2^ from a specialised bloodstream form telomeric expression site (BES), where it is co-transcribed along with 4 or more expression site associated genes (ESAGs) using a single promoter^3–5^. Switching VSG is achieved by switching to transcription of one of several different telomeric BESs^4^ or replacement, by recombination, of the VSG in the active BES with one of the ~2500 VSG gene and pseudogene variants elsewhere in the genome^6^. The active BES is found in a specialised Pol I-containing, non-nucleolar, nuclear structure called the expression site body (ESB)^2^, from which inactive BESs are excluded. The ESB is only present in bloodstream form parasites^7^, despite procyclic forms (in tsetse fly) also employing Pol I-dependent transcription of their invariant surface coat (procyclin). Elegant biochemical candidate approaches and genetic screens of VSG expression have revealed the importance of epigenetic silencing^8^, telomere^9–12^ and chromatin factors^13–20^ and SUMOylation^21,22^. VEX proteins, required for exclusion of the inactive BESs^23,24^, associate the single active BES with the Spliced Leader (SL) array^25^ chromosomal locations. These contain the repetitive genes encoding a sequence which, after transcription and processing, is added to every trypanosome mRNA^26^. Hence, in addition to other properties, VEX proteins link an ESB located exclusion phenomenon to an active VSG gene abundant mRNA processing capability. Notwithstanding these advances, bloodstream-specific factors (Fig. 1A, Extended Data Table 1) remain elusive and the statement that “No ESB-specific factor has yet been identified”^27^ still holds true. Here, we used a medium throughput localisation screen to identify ESB-specific protein 1 (ESB1), which is only expressed in mammalian infectious forms and localised specifically to the ESB. ESB1 is required for VSG expression and is located near the active VSG promoter, with overexpression activating inactive VSG promoters. We show that ESB1 is required for recruitment of some but not all ESB components, revealing the ESB has separately assembled sub-domains. Many trypanosomatid parasites have a divergent ESB1 ortholog and therefore ESB1 potentially represents an important class of trypanosome transcription regulators.

## Results

**Extended Data Fig. 1:**
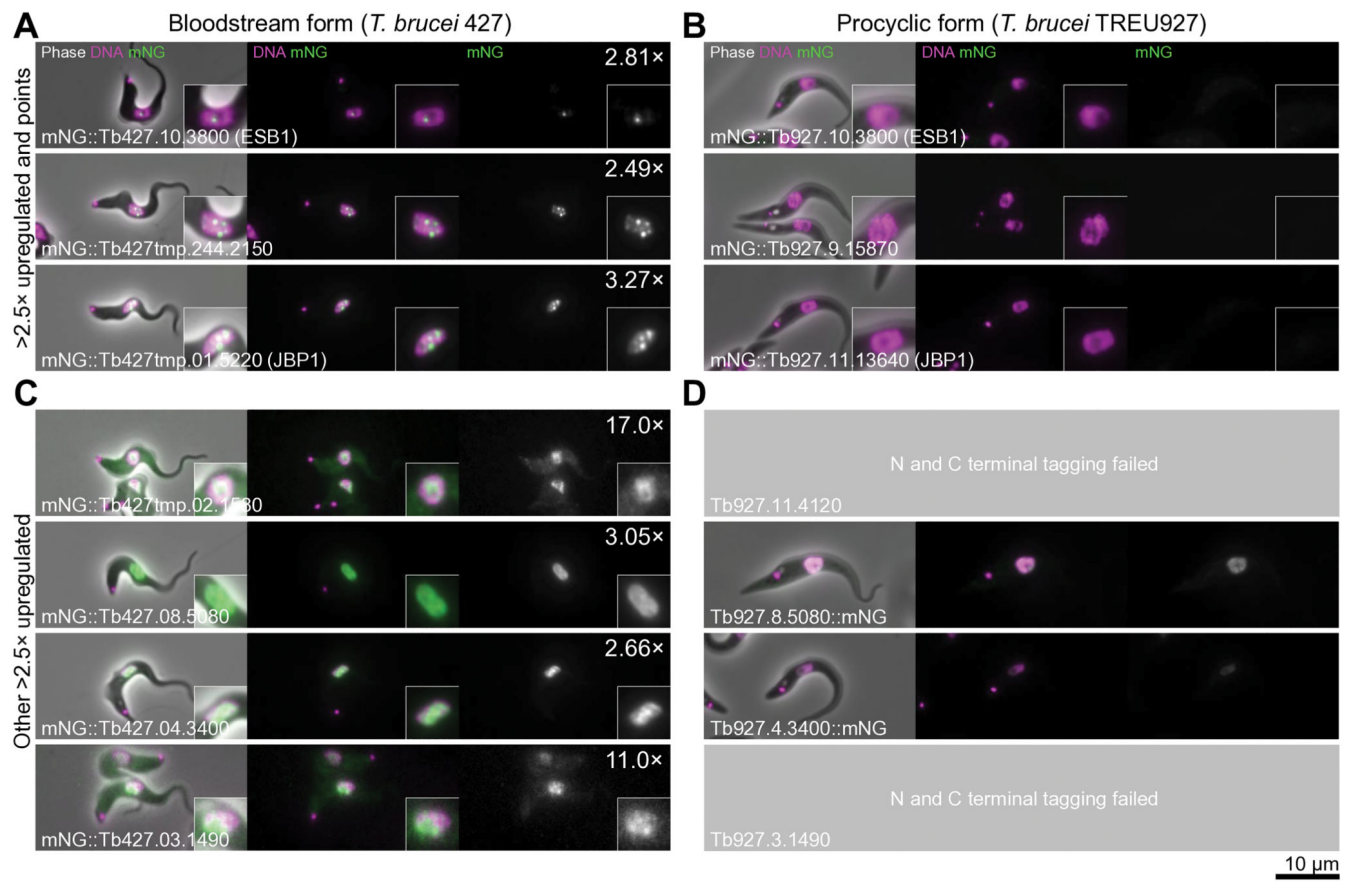
Bloodstream form-upregulated nuclear *T. brucei* proteins. Screening epifluorescence images of cell lines expressing tagged proteins, each image representative of *n* = 1 non-clonal cell line. Images for each cell line are laid in the same format: Left, an overlay of the phase contract (grey), mNG fluorescence (green) and Hoechst DNA stain (magenta). Middle, the DNA stain and the mNG fluorescence. Right, the mNG fluorescence in greyscale. Fold upregulation in bloodstream form cells is shown in the top right. **A)** Subcellular localisation of all 3 proteins >2.5× upregulated in bloodstream forms^49^ which localised to one or multiple points in the nucleus when tagged at the N terminus in bloodstream forms. **B)** Subcellular localisation of the 3 proteins in A) in equivalent procyclic form cell lines, shown at the same contrast levels. **C)** Subcellular localisation of the remaining 4 proteins >2.5× upregulated in bloodstream forms which localised to the wider nucleus when tagged at the N terminus in bloodstream forms. **D)** Subcellular localisation of the 4 proteins in C) in equivalent procyclic form cell lines, from TrypTag^28^, shown at the same contrast levels. We were unable to generate two cell lines.

**Extended Data Fig. 2:**
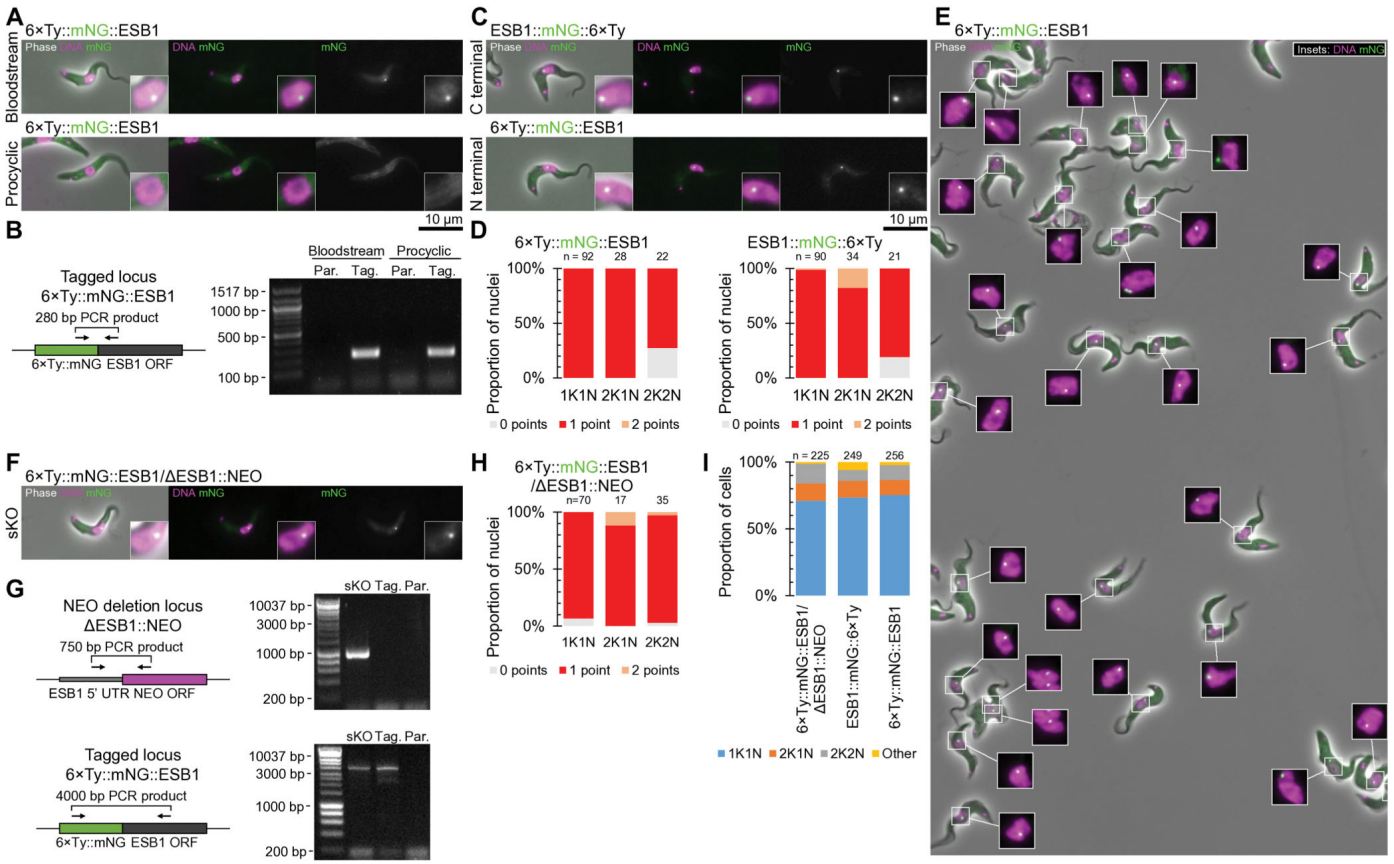
Tagging does not perturb ESB1 localisation or function. **A)** Clonal bloodstream form and procyclic form cell lines expressing Tb427.10.3800 or Tb927.10.3800 (ESB1) N terminally tagged with 6×Ty::mNG respectively were re-generated following the initial screen. Epifluorescence images representative of *n* = 1 clonal cell line of the localisation of the tagged protein by mNG fluorescence. **B)** Confirmation of the expected genetic modification of the cell lines in A) by PCR from genomic DNA using a forward mNG and a reverse ESB1 ORF primer. Schematic shows the primer binding sites, uncropped DNA gel shows the resulting PCR products from extracted genomic DNA from the tagged (Tag.) or parental (Par.) cell line. **C)** Epifluorescence images representative of *n* = 1 clonal cell line of bloodstream form cell lines expressing 6×Ty::mNG::ESB1 (N terminal tag), ESB1::mNG::6×TY (C terminal tag). **D)** Count of the number of points per nucleus at different stages of the cell cycle (1K1N, 2K1N and 2K2N) for N or C terminally tagged ESB1, from *n* = 1 replicate. **E)** Example field of view representative of *n* = 1 clonal cell line of cells expressing 6×Ty::mNG::ESB1 showing the strictly singular nature of the ESB1 nuclear focus. **F)** Epifluorescence image representative of *n* = 1 clonal cell line of a single knockout (sKO) bloodstream form cell line with one N terminally tagged ESB1 allele and the other deleted by replacement with a drug selectable marker. **G)** PCR validation of the sKO cell line. Schematics represent the deleted ESB1 ORF (top) and N terminally tagged locus (bottom) and primer binding sites, uncropped DNA gels shows the resulting PCR products from extracted genomic DNA. **H)** Count of the number of points per nucleus at different stages of the cell cycle for the sKO cell line, from *n* = 1 replicate. **I)** Proportion of cells at different stages of the cell cycle for the sKO in comparison to N or C terminal tagging, from *n* = 1 replicate, no changes *p* ≤ 0.05 from χ^2^ test.

**Fig. 1:**
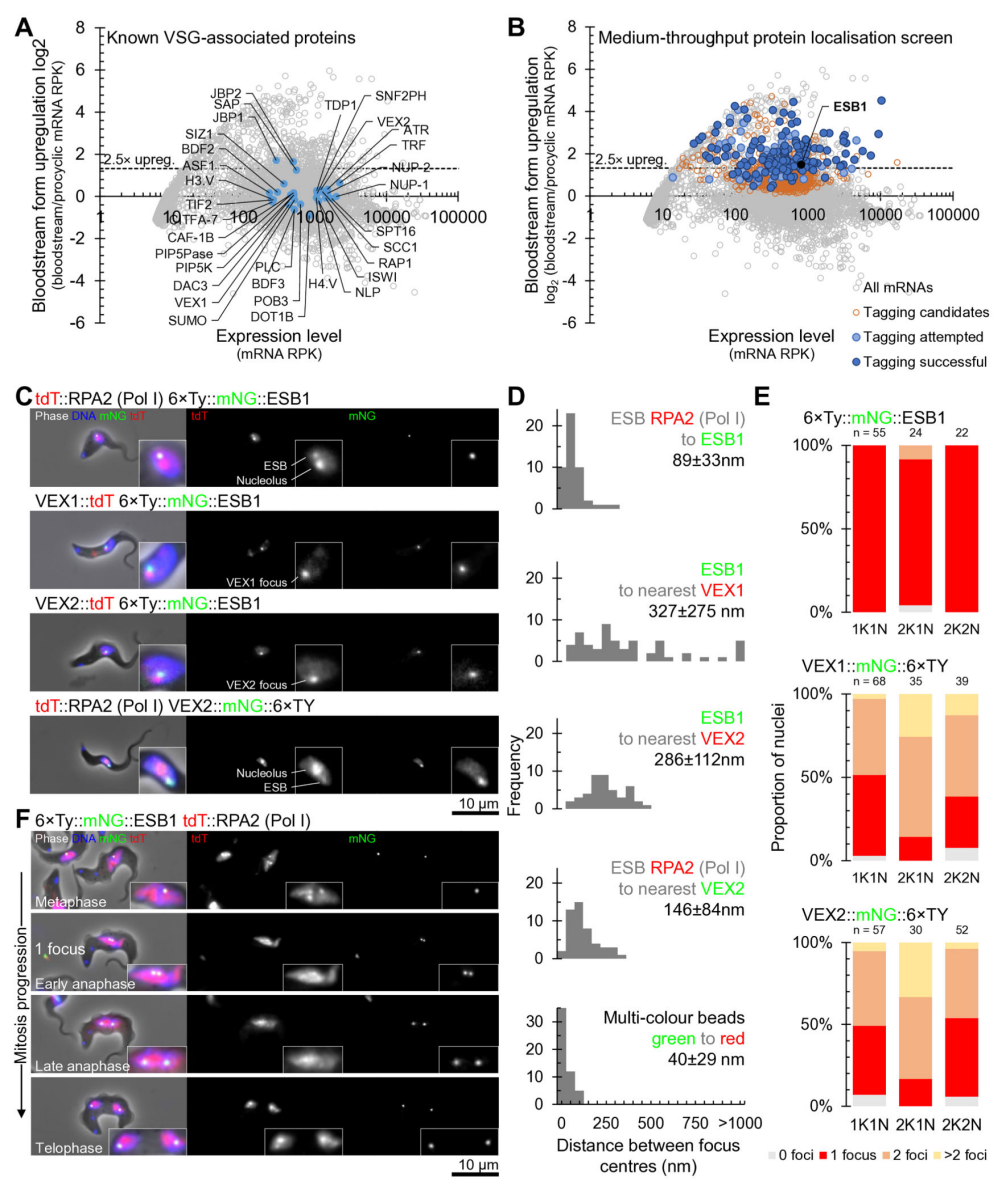
A protein localisation screen identified ESB1. **A,B)** Degree of upregulation of *T. brucei* mRNAs in bloodstream forms previously determined by RNAseq^49^ highlighting **A)** known VSG monoallelic expression-associated factors and **B)** candidates for tagging we selected, attempted and successfully localised. **C-F)** Fluorescence microscopy analysis of ESB1 subcellular localisation relative to known ESB-associated proteins. **C)** Representative images from at least *n* = 3 independent sample preparations of G1 (1K1N) cells from cell lines expressing one mNeonGreen-tagged (mNG) and one tdT-tagged ESB-associated protein. For cells expressing VEX1 or VEX2, examples with one nuclear focus are shown. **D)** Histograms of pairwise distance measurements between the ESB1 focus, RPA2 ESB focus and the nearest VEX1 or VEX2 focus. For each, *n* ≥ 45 cells from one clonal cell line, all distances are significantly different (*p* < 10^-80^, two-tailed Mann-Whitney U test). Multi-colour beads are a control for measurement accuracy (true distance of zero). **E)** Number of mNG-tagged ESB1, VEX1 or VEX2 foci per nucleus in different cell cycle stages, *n* indicates number of cells counted from one clonal cell line. The number of ESB1 foci significantly differs from the number of VEX1 or VEX2 in 1K1N cell nuclei (*p* < 10^-9^, χ^2^ test). **F)** ESB1 localisation in mitotic nuclei representative of *n* = 3 independent sample preparations.

We performed a candidate protein localisation screen of proteins upregulated in the bloodstream form^28^ of unknown function, and identified an ESB-specific protein. G1 bloodstream form nuclei have one extranucleolar ESB^7,29^. From 207 candidates, 153 were successfully localised and only one (Fig. 1B), Tb427.10.3800, exhibited this localisation (Fig. 1C, Extended Data Fig. 1A) whilst endogenous tagging in the procyclic form gave no detectable signal (Extended Data Fig. 1B, Extended Data Fig. 2A,B). We named this protein ESB1.

We used well-characterised ESB markers to confirm the ESB1 localisation. Pol I is the founding component of the ESB and localises to both the nucleolus and the ESB in bloodstream forms^7^. ESB1 lies extremely close to Pol I (RPA2) at the ESB (Fig. 1C) confirmed by measurement of the distance between signal centre points (Fig. 1D). The ESB also has a VEX sub-complex involved in exclusion of inactive ESs^23^. ESB1 lies ~300 nm from the nearest VEX1 or VEX2 focus (Fig. 1C,D), significantly further than Pol I to VEX2 (Fig. 1D)^24^, suggesting the centre of the Pol I body lies between ESB1 and VEX2. After S phase, cells still exhibit a single ESB before the nucleus undergoes closed mitosis. Unlike VEX1 and VEX2, ESB1 always localises to a single focus per nucleus (Fig. 1E), whether tagged at the N or C terminus (Extended Data Fig. 2C-E). As *T. brucei* are we also confirmed, by deletion of the untagged allele, that expression of N terminally-tagged ESB1 in the absence of the wild-type allele gave the same localisation (Extended Data Fig. 2F-H) and we saw no morphological or cell growth defect (Extended Data Fig. 2I). Similar to the ESB Pol I signal^29^ a second ESB1 focus only forms during anaphase (Fig. 1F). ESB1 is therefore specific to the ESB both spatially (localisation) and temporally (life cycle stage-specific expression and cell cycle-dependent localisation).

### ESB1 is necessary for active ES transcription

**Extended Data Fig. 3:**
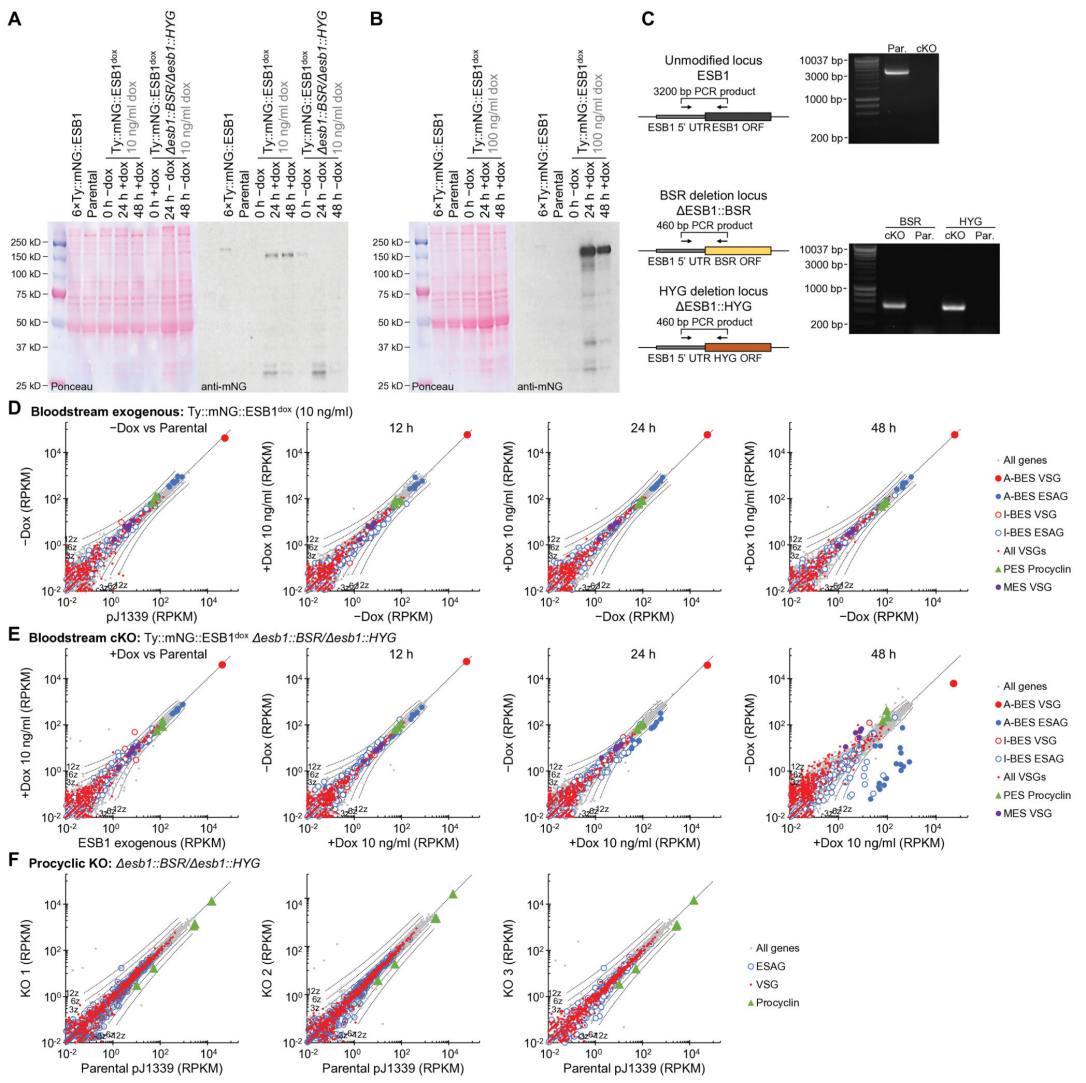
Generation and validation of an ESB1 conditional knockout. **A)** Western blot validation of the cKO cell line and the BSF pDex577 tagged ESB1 exogenous expression cell line (the intermediate in cKO generation), both induced with 10 ng/ml doxycycline. Predicted molecular weights for ESB1 are: 108 kDa (untagged), 137 kDa (Ty::mNG tag) and 145 kDa (6×Ty::mNG tag). The uncropped Ponceau-stained membrane and anti-mNG blot are shown. **B)** Western blot validation of the BSF pDex577 tagged ESB1 exogenous expression cell line induced with 100 ng/ml doxycycline for overexpression. **C)** Validation of genetic modifications of the ESB1 conditional knockout (cKO). Schematics represent the deleted and tagged loci and primer binding sites and orientations, uncropped DNA gels shows the resulting PCR products from extracted genomic DNA. **D)** mRNA abundance in the BSF pDex577 tagged ESB1 exogenous expression cell line, plotting RPKM of uniquely mapped RNAseq reads. The exogenous expression prior to addition of doxycycline (0 h) is plotted relative to the parental pJ1339 cell line. Other plots are 12, 24 and 48 h after addition of 10 ng/ml doxycycline relative to the cell line grown without doxycycline. Each shows *n* = 1 induction replicate. **E)** mRNA abundance in the cKO, plotting RPKM of uniquely mapped RNAseq reads. The cKO prior to doxycycline washout (0 h) is plotted relative to the BSF pDex577 tagged ESB1 exogenous expression cell line induced with 10 ng/ml doxycycline. Other plots are 12, 24 and 48 h after doxycycline washout relative to the cell line grown with 10 ng/ml doxycycline. Each shows *n* = 1 induction replicate. **F)** mRNA abundance in the procyclic form KO, plotting RPKM of uniquely mapped RNAseq reads for three clonal KO cell lines relative to the parental cell line.

**Fig. 2:**
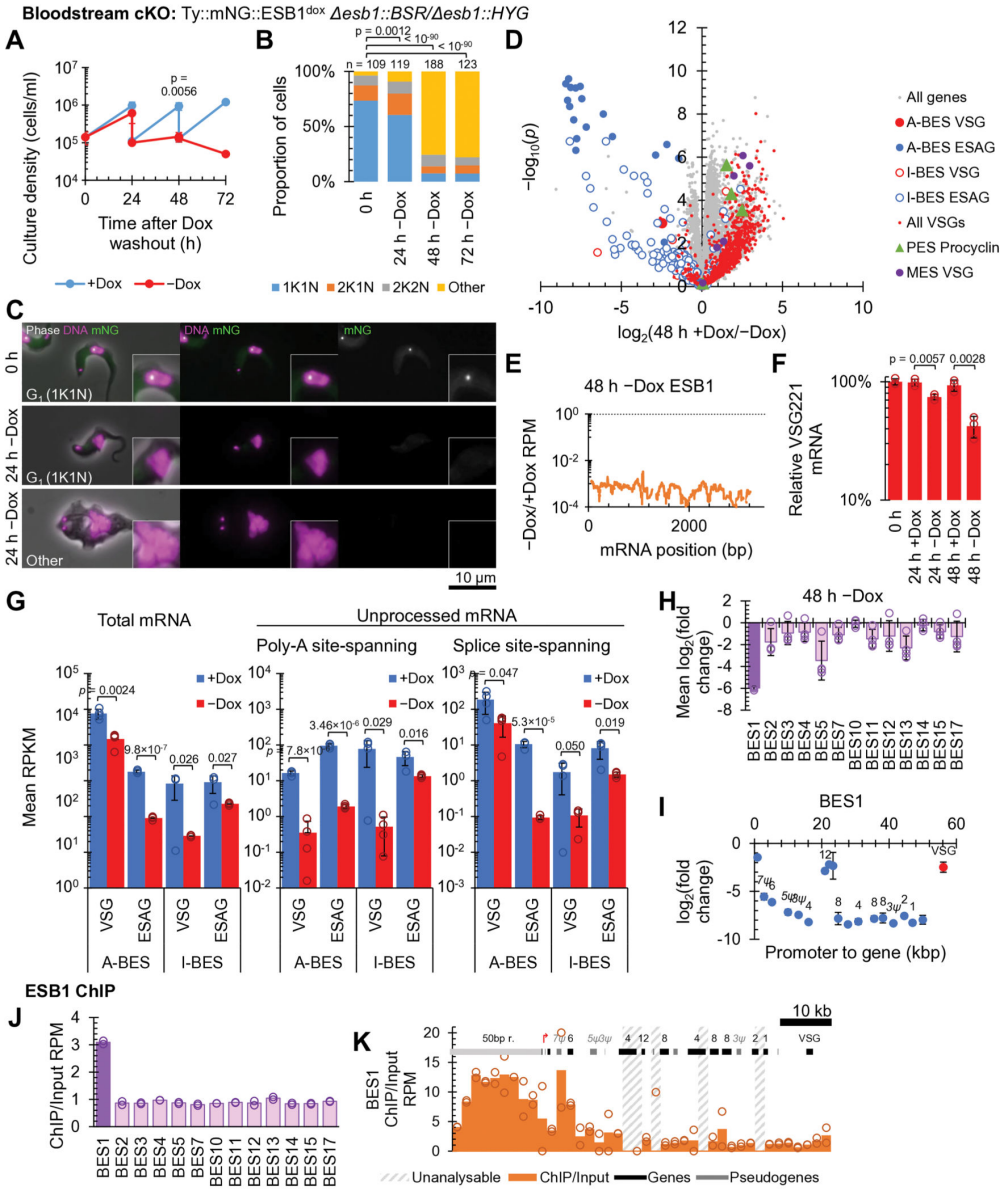
ESB1 is vital for bloodstream forms and required for transcription from the active VSG expression site. **A-F)** Cellular phenotype of bloodstream form ESB1 cKO. Exogenous mNG-tagged ESB1 expression was maintained with 10 ng/ml doxycycline (+Dox) in the bloodstream form ESB1 cKO (cell line validation in Extended Data Fig. 3A-D) and doxycycline washout (−Dox) induced the cKO phenotype. **A)** Culture growth (with subculture), mean ± SD, *n* = 3 inductions. *p* shown at 48 h, two-tailed t test, log cumulative growth. **B)** Counts of morphologically abnormal (‘Other’) cells following washout. *n* indicates number of cells counted, representative example from *n* = 3 inductions. *p* from χ^2^ test. **C)** Representative images from *n* = 3 independent inductions showing mNG-tagged ESB1 signal before and after 24 h −Dox. **D)** Volcano plot of change in mRNA abundance determined by RNAseq 48 h −Dox, *n* = 4 inductions (further time points in Extended Data Fig. 3E). *p* from two-tailed t test. A-BES and I-BES indicate active and inactive BES respectively. **E)** Change in ESB1 ORF read coverage 48 h −Dox, mean of *n* = 4 inductions. **F)** qRT-PCR quantitation of the A-ES VSG mRNA (VSG221) −Dox, mean ± SD from *n* = 3 inductions. *p* ≤ 0.05 shown from two-tailed t test. **G-I)** Profile of transcript abundance change on ESB1 loss in bloodstream forms. **G)** Changes to total and unprocessed mRNA grouped into A-BES or I-BES VSG(s) and ESAGs for the cKO +/−Dox. Mean ± SD from *n* = 4 inductions. *p*≤ 0.05 shown from two-tailed t test. **H)** Average change in transcript abundance averaged per BES and **I)** per gene for the active BES plotted by distance from the promoter 48 h −Dox. *n* = 4 inductions, mean ± SD. **J,K)** ESB1 ChIPseq shown as the ratio of ChIP to input DNA, plotting **J)** mean ratio per BES and **K)** mean ratio in 2 kb bins across the active BES (extended in Extended Data Fig. 4). Unanalysable bins had insufficient uniquely-mapped reads from the input DNA. *n* = 2 replicates.

To determine ESB1 function, we generated a bloodstream form ESB1 conditional knockout (cKO) cell line (Extended Data Fig. 3A-E). ESB1 cKO gave undetectable levels of ESB1 protein by 24 h (Extended Data Fig. 3A) which caused a profound proliferation defect due to failure of cytokinesis and further rounds of organelle duplication (Fig. 2A-C). To detect any effect on BES transcription, we used RNAseq to profile mRNA levels which showed ESB1 cKO caused a dramatic decrease (~250×) in ESAG mRNAs, predominantly those transcribed from the active BES (Fig. 2D, Extended Data Fig. 3E), associated with almost total loss of ESB1 transcript (Fig. 2E). mRNAs from the VSG gene in the active BES decreased ~8× (Fig. 2D) which we confirmed by qRT-PCR (Fig. 2F). The smaller decrease in VSG mRNAs is likely explained by the longer half-life of VSG mRNAs^30^.

To understand at what stage ESB1 functions in VSG and ESAG mRNA production we analysed changes to nascent mRNAs in the bloodstream form ESB1 cKO. Co-transcriptional trans-splicing and polyadenylation generate mature mRNAs^31^, enabling quantification of unprocessed transcript from RNA sequencing reads spanning the spliced leader acceptor (SLAS) and polyadenylation (PAS) sites. Unprocessed ESAG and VSG mRNAs also dropped dramatically following ESB1 cKO (Fig. 2G), indicating ESB1 cKO reduces active BES transcription rather than mRNA processing. Some low-processivity transcription of inactive ESs occurs^32,33^. ESB1 cKO caused a small reduction in unprocessed transcript from inactive ESs (Fig. 2G) and mRNAs transcribed from specific inactive ESs (Fig. 2H), while mRNAs from promoter-proximal ESAG genes in the active ES tended to be less strongly reduced (Fig. 2I). Therefore, the highly processive active ES transcription is ESB1-dependent, with ESB1 cKO leaving a little residual transcription like silent ESs.

**Extended Data Fig. 4:**
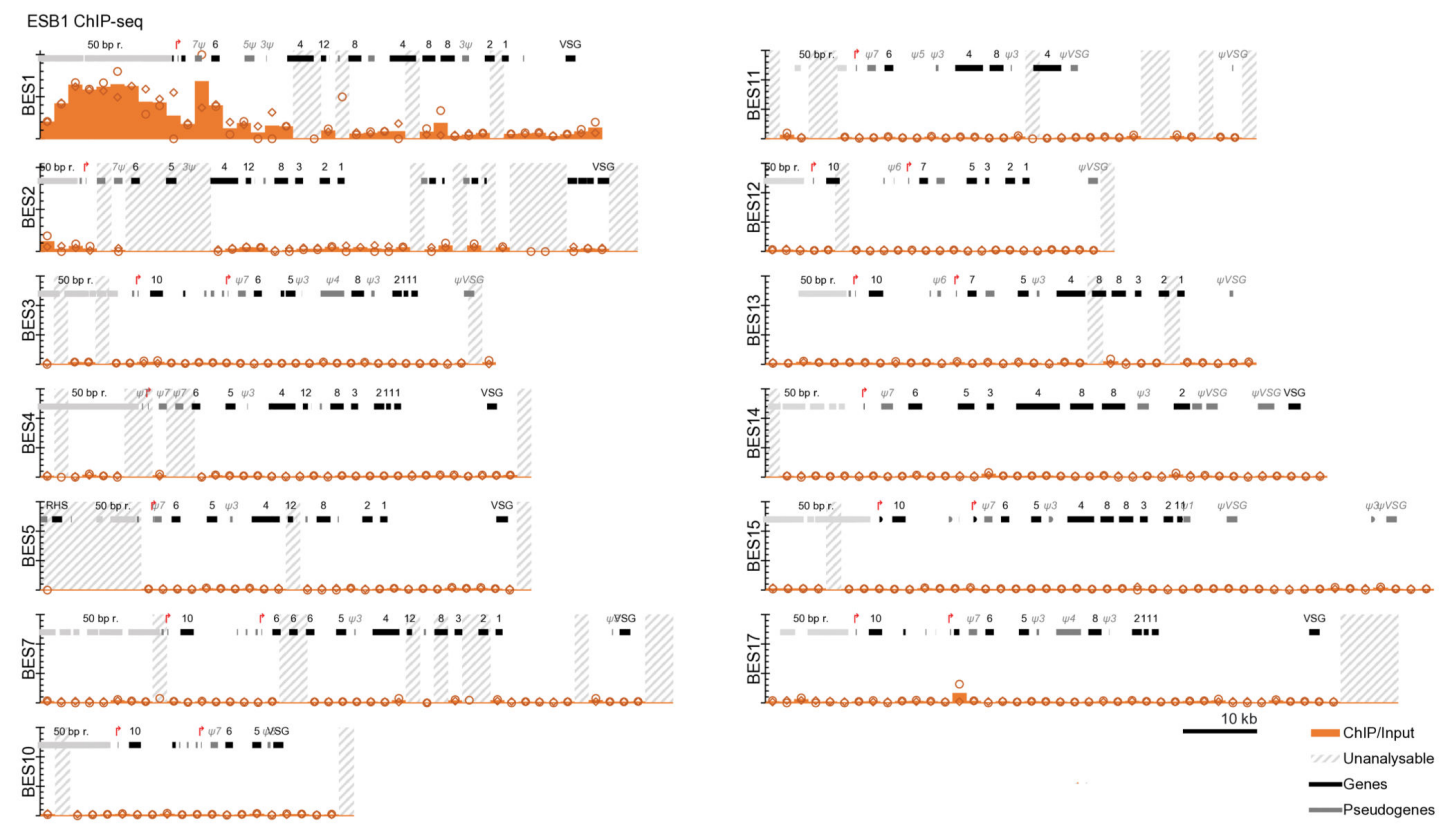
Extended presentation of ChIP data showing the active and inactive BESs. ESB1 ChIPseq in BSFs shown as the ratio of ChIP to input DNA in 2 kb bins across the active BES (BES1) and all inactive BESs. Unanalysable bins had insufficient uniquely-mapped reads from the input DNA. Extended version of Fig. 2K. *n* = 2 replicates, data points represent individual replicates and bars represent the mean.

A specific transcription activator would be predicted to associate only with the promoter region of the active ES. Therefore, we carried out ESB1 chromatin immunoprecipitation sequencing (ChIPseq). Across the genome, the highest peak in ESB1 ChIP/input DNA ratio (30× background signal) was in the active ES. Among the ESs the active ES had the highest average ChIP ratio (Fig. 2J, Extended Data Fig. 4), due to a large peak between ~5 and 15 kb upstream and a smaller peak ~5 kb downstream of the Pol I promoter (Fig. 2K). The former corresponds to the imperfect 50 bp repeats found upstream of all ESs^34,35^, but ESB1 only associates with these repeats at the active ES.

**Fig. 3:**
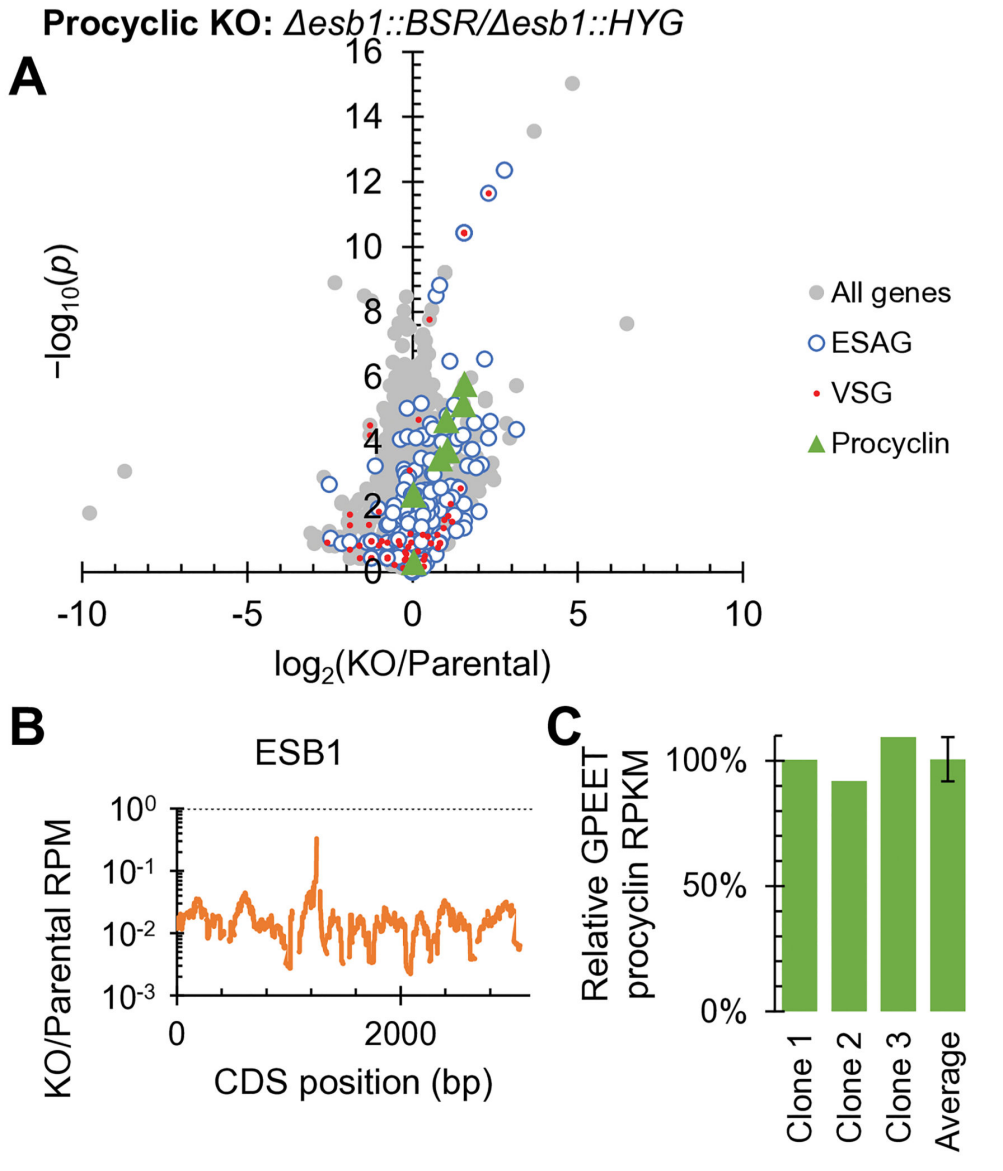
ESB1 is dispensable in procyclic forms. mRNA abundance phenotype of procyclic form ESB1 KO. **A)** Volcano plot of change in mRNA abundance, *n* = 3 independent clonal cell lines (plotted individually in Extended Data Fig. 3F). *p* from two-tailed t test. **B)** Change in ESB1 ORF read coverage, mean of *n* = 3 clonal ESB1 KO cell lines. **C)** Abundance of GPEET procyclin in *n* = 3 clonal ESB1 KO cell lines relative to the parental cell line determined by RNAseq. Fourth bar shows mean ± SD.

Procyclic forms lack an active BES, an ESB and do not express ESB1, although they use Pol I for expression of their surface coat protein (procyclin) whose locus we refer to as a procyclin expression site (PES). We tested ESB1 cryptic function in procyclic forms by deleting both ESB1 alleles, which gave no apparent growth or morphology defect. RNAseq confirmed normal high expression of GPEET procyclin and no major changes to other mRNA transcripts (Fig. 3, Extended Data Fig. 3F). ESB1 is therefore vital in bloodstream forms for monoallelic VSG expression but is dispensable in procyclic forms.

**Extended Data Fig. 5:**
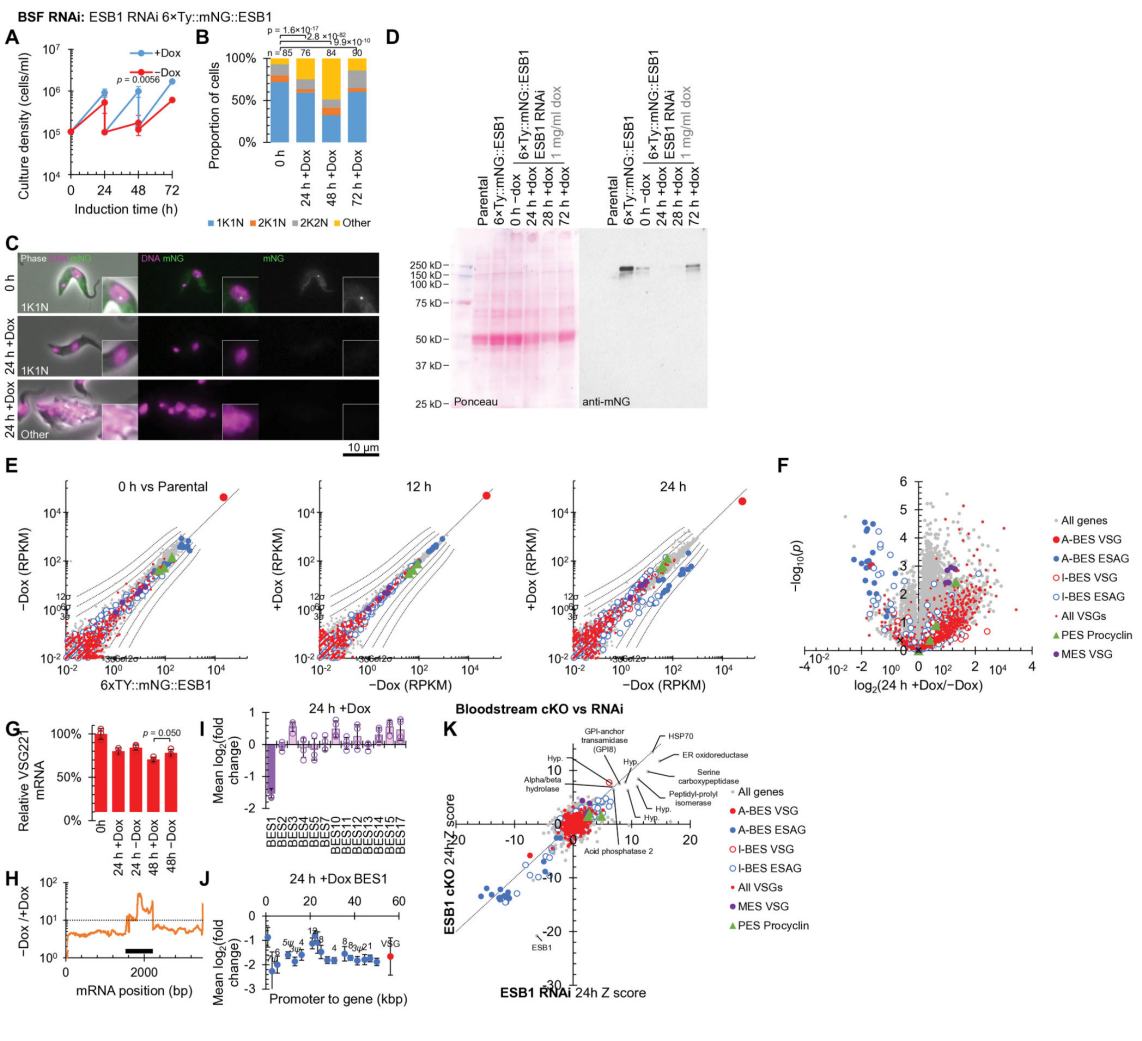
RNAi knockdown confirms ESB1 is vital and required for active BES transcription. Cellular phenotype of ESB1 RNAi knockdown. **A)** Growth curve of the ESB1 RNAi following doxycycline induction (+Dox) in comparison to uninduced (−Dox), using repeated subculture to maintain culture density under ~1×10^6^ cells/ml. Mean ± SD from *n* = 3 inductions. *p* shown at 48 h, two-tailed t test, log cumulative growth. **B)** Number of kinetoplasts (K) and nuclei (N) per cell, counted by light microscopy, at 24 h intervals following washout of doxycycline from the ESB1 cKO. representative example from *n* = 3 inductions. *p* from χ^2^ test. 1K1N, 2K1N and 2K2N are normal cell cycle stages. **C)** Representative images from *n* = 3 independent inductions of the ESB1 RNAi cell line before and after induction, showing a morphologically normal (1K1N) cell and a typical abnormal cell after 24 h induction. mNG signal is not detectable after 24 h induction. **D)** Uncropped anti-mNG Western blot validation of the ESB1 RNAi cell line. **E)** mRNA abundance in the BSF ESB1 RNAi cell line, plotting RPKM of uniquely mapped RNAseq reads. The uninduced cell line (0 h) is plotted relative to the parental 6×Ty∷mNG::ESB1 cell line. Other plots are 12 and 24 after addition of 1 mg/ml doxycycline relative to the cell line grown without doxycycline. Each represents *n* = 1 induction replicate. **F)** Volcano plot of change mRNA abundance determined by RNAseq 24 h +Dox, *n* = 4 inductions. *p* from two-tailed t test. A-BES and I-BES indicate active and inactive BES respectively. **G)** qRT-PCR measurement of active BES VSG mRNA relative to the parental cell line, mean ± SD from *n* = 3 inductions, *p* ≤ 0.05 shown from two-tailed t test. **H)** Change in RNAseq read coverage over the ESB1 open reading frame shows reduced ESB1 transcript, mean from *n* = 4 inductions. **I)** Average change in transcript abundance averaged per BES and **J)** per gene for the active BES plotted by distance from the promoter 48 h −Dox, *n* = 4 inductions, mean ± SD from *n* = 4 inductions. **K)** Correlation of per mRNA Z scores for the bloodstream ESB1 RNAi cell line with the ESB1 cKO after 24h induction. Upregulated genes are labelled, and are ER stress associated.

For further experiments, the bloodstream form cKO phenotype was recapitulated with the more experimentally amenable RNAi knockdown of ESB1 (Extended Data Fig. 5). mRNA abundance changes correlated extremely well with cKO (Fig. 2, Extended Data Fig. 5K), with the same ESAGs and VSGs mRNAs reduced, as were the same set of 11 upregulated mRNAs (likely ER stress-associated, Extended Data Fig. 5K). The rapid lethality of the RNAi phenotype naturally led to appearance of RNAi escape sub-populations^36^; therefore, we analysed only early RNAi time points.

### ESB molecular composition depends on ESB1

**Fig. 4:**
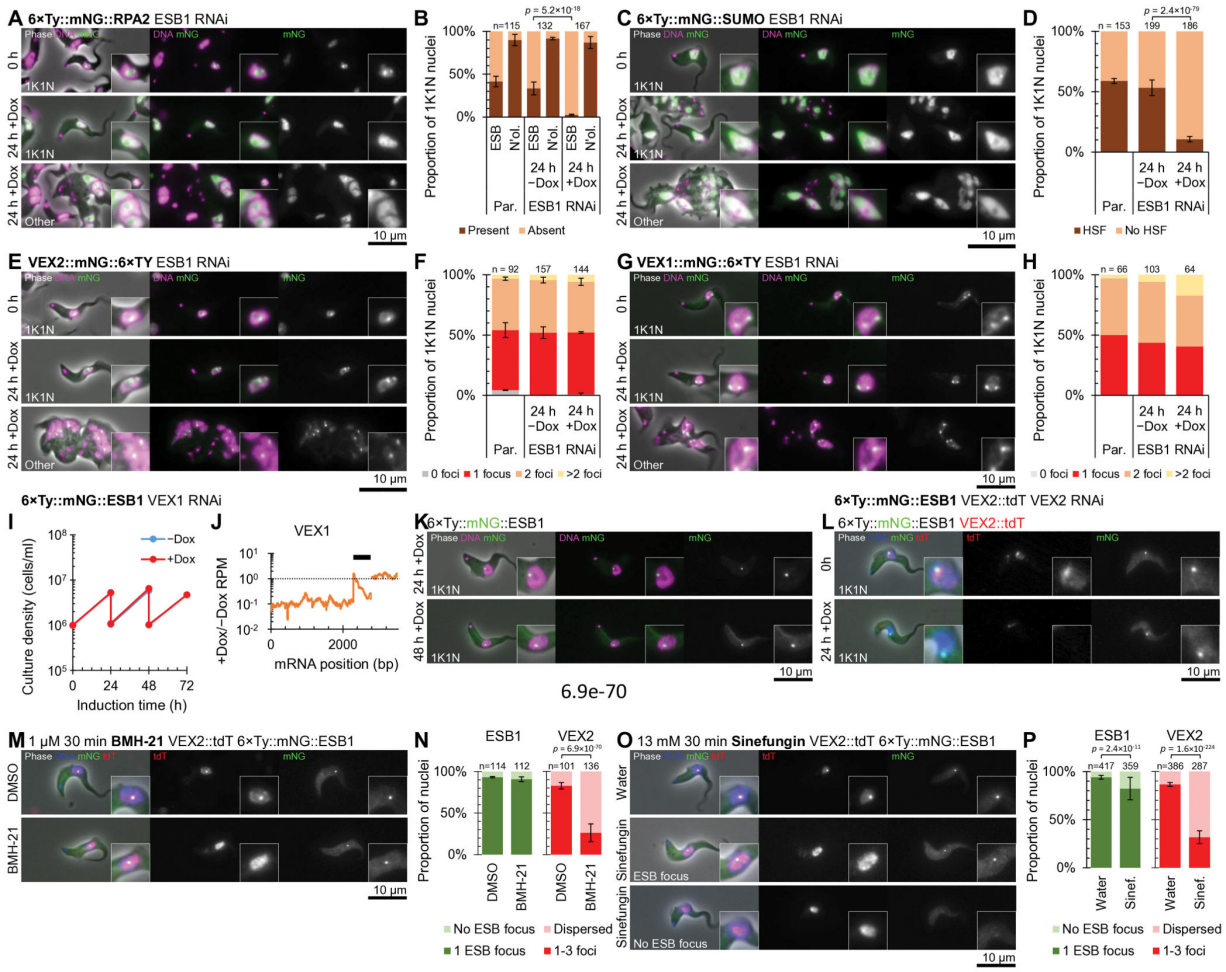
ESB1 is required for formation of a subset of ESB substructures. **A-H)** Effect of doxycycline-inducible ESB1 RNAi knockdown (knockdown characterised in Extended Data Fig. 5) on mNG-tagged **A-B)** RPA2, **C-D)** SUMO, **E-F)** VEX2 and **G-H)** VEX1 localisation. Each cell line was maintained without doxycycline (−Dox) then induced with 1 mg/ml doxycycline (+Dox). **A,C,E,G)** Representative fluorescence images from *n* = 3 (except for **G**, *n* = 1) independent inductions of tagged protein localisation in morphologically normal and abnormal cells after 24 h RNAi induction. **B,D,F,H)** Counts of the number of cells **B)** with an RPA2-containing ESB focus and an RPA2-containing nucleolus (N’ol.), **D)** with a highly SUMOylated focus (HSF), **F)** the number of VEX2 foci or **H)** the number of VEX1 foci in comparison to the parental (Par., no RNAi) cell line. mean ± SD from 3 independent inductions (except for **H**, *n* = 1), *n* indicates total number of cells. *p* ≤ 0.05 shown from χ^2^ test. **I-K)** Effect of doxycycline-inducible VEX1 RNAi knockdown on mNG-tagged ESB1 localisation. **I)** Culture growth (with subculture) and **J)** change in VEX1 ORF read coverage showing effective knockdown determined by RNAseq, *n* = 1 induction. **K)** Representative fluorescence images from *n* = 1 induction showing mNG-tagged ESB1 localisation after VEX1 knockdown. **L)** Representative fluorescence images from *n* = 1 induction showing mNG-tagged ESB1 and tdT-tagged VEX2 localisation after VEX2 knockdown. **M-P)** Effect of **M,N)** 1 μM BMH-21 or **O,P)** 13 mM sinefungin on ESB1 and VEX2 localisation. **M,O)** Example fluorescence microscope images following 30 min solvent control (DMSO or water) or compound treatment. **N,P)** Counts of the number of cells with focused or dispersed ESB1 and VEX2. Mean ± SD from 3 replicates, *n* indicates total number of cells, *p* ≤ 0.05 shown from χ^2^ test.

We next determined whether ESB1, and thus active ES transcription, is required for the normal molecular composition of the ESB. We generated a panel of cell lines carrying the inducible ESB1 RNAi construct and tagged ESB-associated proteins: RPA2, SUMO (as the ESB is associated with a highly SUMOylated focus^21^), VEX1 or VEX2 (Fig. 4A-H). As shown by others, the ESB focus of RPA2 was visible in 40% of G1 nuclei (i.e. when not occluded by nucleolar RPA2)^7^ and the highly SUMOylated focus in ~60% of G1 nuclei^21^. After 24 h induction of ESB1 RNAi, RPA2 and SUMO were more dispersed through the nucleus and fewer nuclei had an ESB focus, both in morphologically normal and abnormal cells, while nucleolar RPA2 was unaffected (Fig. 4A-D). As seen previously, VEX1 and VEX2 localised to 1 or 2 foci in the nucleus. After 24 h induction of ESB1 RNAi the localisation pattern was unchanged, both in morphologically normal and abnormal cells (Fig. 4E-H). This indicates ESB1 is necessary for both recruitment of Pol I to the ESB and higher local SUMOylation to form the HSF, but not formation of VEX foci.

**Extended Data Fig. 6:**
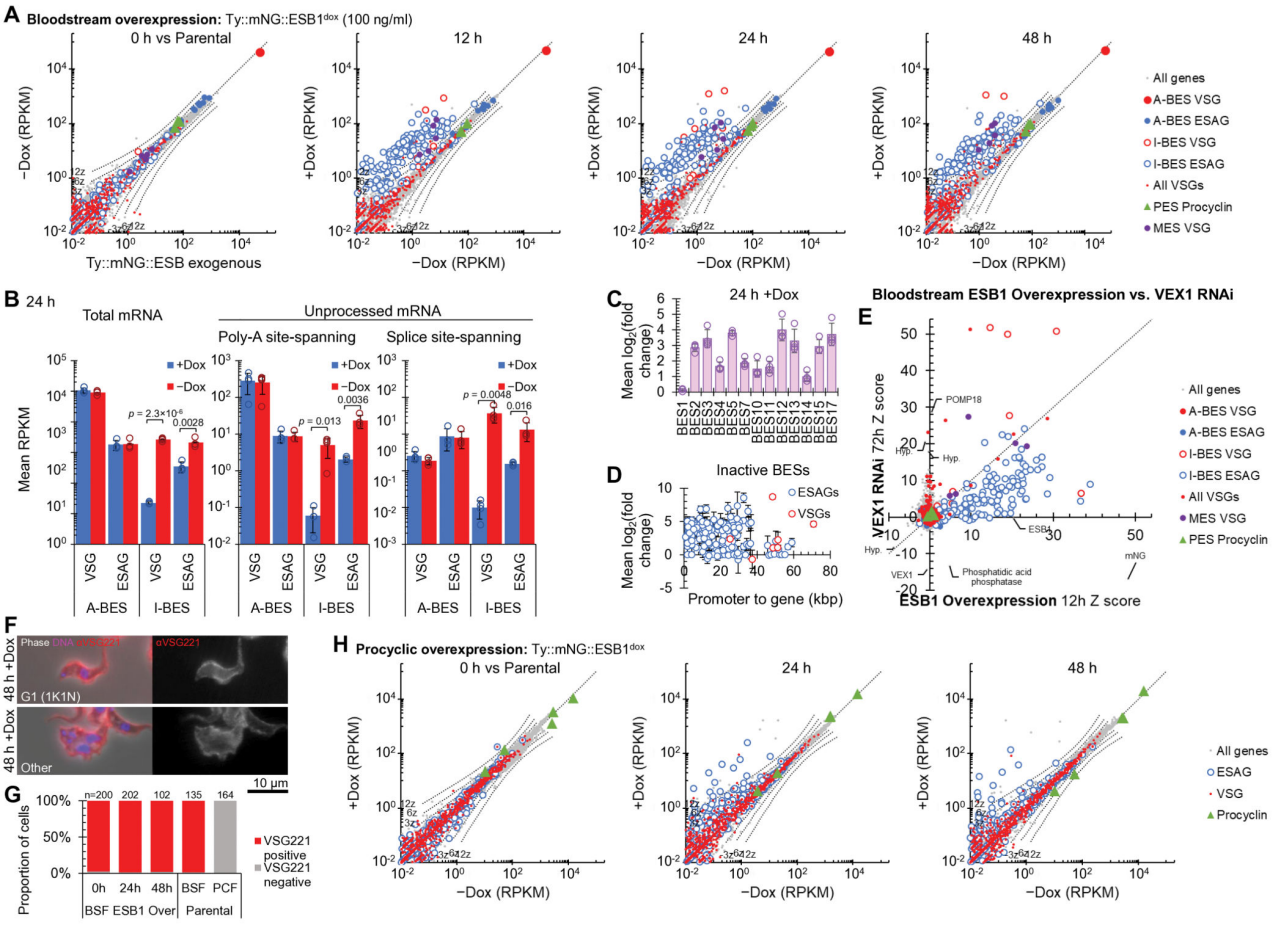
Extended analysis of the bloodstream and procyclic form overexpression analysis. **A)** mRNA abundance in the bloodstream form ESB1 overexpression cell line, plotting RPKM of uniquely mapped RNAseq reads. The overexpression prior to doxycycline addition (0 h) is plotted relative to the parental pJ1339 cell line. Other plots are 12, 24 and 48 h after 100 ng/ml doxycycline addition relative to the cell line grown without doxycycline. Each represents *n* = 1 induction replicate. **B)** Changes to total and unprocessed (not polyadenylated or not spliced) mRNA grouped into A-BES or I-BES VSG(s) and ESAGs for the overexpression +/−Dox. Mean ± SD from *n* = 4 inductions. *p*≤ 0.05 shown from two-tailed t test. **C)** Average change in transcript abundance averaged per BES and **D)** per gene for the inactive BESs plotted by distance from the promoter 12 h +Dox, *n* = 4 inductions, mean ± SD. **E)** Correlation of per mRNA Z scores for the ESB1 overexpression, 12 h induction, with VEX1 RNAi, 72 h induction. Non-VSG and ESAG outliers are labelled. **F,G)** Anti-VSG221 immunofluorescence of the ESB1 overexpression line showing **F)** images of an example morphologically normal and abnormal cell after 48 h overexpression and **G)** counts of the proportion of VSG221-positive cells in comparison to the BSF and PCF cell lines. *n* numbers indicate number of cells counted from 1 induction replicate, no BSF changes *p* ≤ 0.05 from χ^2^ test. **H)** mRNA abundance in the procyclic form ESB1 overexpression cell line, plotting RPKM of uniquely mapped RNAseq reads. The overexpression prior to doxycycline addition (0 h) is plotted relative to the parental pJ1339 cell line. Other plots are 12, 24 and 48 h after 1 mg/ml doxycycline addition relative to the cell line grown without doxycycline. Each represents *n* = 1 induction replicate.

The inverse, whether the ESB1 focus is VEX1 or VEX2-dependent, was analysed by their depletion using RNAi and observing tagged ESB1 (Fig. 4I-L). VEX1 knockdown was confirmed using RNAseq profiling of mRNA and, as previously described^23^, we saw derepression of inactive BESs (Extended Data Fig. 6E) with no growth defect (Fig. 4I,J). VEX2 knockdown was confirmed by carrying out knockdown in a cell line expressing tagged VEX2. ESB1 localisation was unchanged on either VEX1 or VEX2 knockdown (Fig. 4K,L); therefore, formation of a singular ESB is not dependent on repression of inactive BESs by the VEX complex. The ESB1 and VEX2 compartments also have differing sensitivity to small molecule inhibitors. VEX2 foci became distributed following inhibition of Pol I transcription (BMH-21, an indirect Pol I inhibitor acting via DNA binding, Fig. 4M,N) or splicing (sinefungin, Fig. 4O,P), while the ESB1 focus was not strongly affected by either.

### ESB1 overexpression activates transcription from silent ESs

**Fig. 5:**
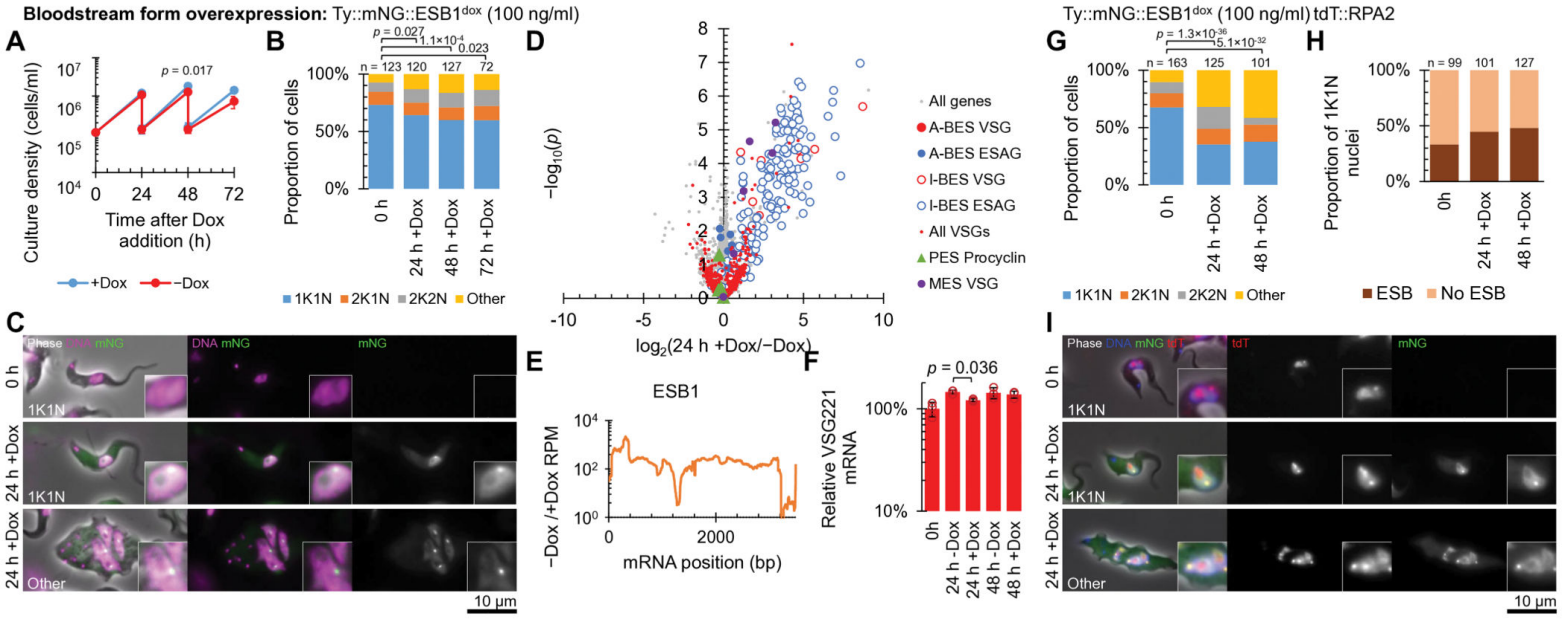
ESB1 overexpression in BSFs activates inactive BESs without affecting ESB formation. **A-F)** Cellular phenotype of mNG-tagged ESB1 overexpression in bloodstream forms induced with 100 ng/ml doxycycline (+Dox). **A)** Culture growth (with subculture), mean ± SD, *n* = 3 inductions. *p* shown at 48 h, two-tailed t test, log cumulative growth. **B)** Counts of morphologically abnormal (‘Other’) cells following washout. *n* indicates number of cells counted, representative example from *n* = 3 inductions. *p* from χ^2^ test. **C)** Representative images from *n* = 3 independent inductions showing mNG-tagged ESB1 signal before and after 24 h +Dox. **D)** Volcano plot of change in mRNA abundance determined by RNAseq 24 h −Dox, *n* = 4 inductions (further time points in Extended Data Fig. 6A). *p* from two-tailed t test. A-BES and I-BES indicate active and inactive BES respectively. **E)** Change in ESB1 ORF read coverage determined by RNAseq 24 h after washout, mean of *n* = 4 inductions. **F)** qRT-PCR quantitation of the A-ES VSG mRNA (VSG221) +Dox, mean ± SD from *n* = 3 inductions. *p* ≤ 0.05 shown from two-tailed t test. **G-I)** Effect of mNG-tagged ESB1 overexpression on tdT-tagged RPA2 localisation. Counts of **G)** morphologically abnormal cells, **H)** the number of cells with an RPA2-containing ESB focus. *n* indicates number of cells counted from 1 induction, *p* ≤ 0.05 shown from χ^2^ test. **I)** Representative images from *n* = 1 replicate showing tagged protein localisation before induction and in morphologically normal and abnormal cells after ESB1 overexpression.

We then asked whether ESB1 overexpression could force ectopic BES expression and/or supernumerary ESB formation. Significant overexpression was achieved using a cell line with an additional inducible tagged ESB1 locus (using 100 ng/ml doxycycline) (Fig. 5A-F, Extended Data Fig. 3B). In contrast to ESB1 cKO, overexpression gave a small growth reduction and some cytokinesis defects (Fig. 5A-C). Overexpressed ESB1 still localised to the ESB, although with more dispersion in the nucleoplasm and cytoplasm, in both morphologically normal and abnormal cells (Fig. 5C). ESB1 overexpression in a cell line expressing tagged RPA2 showed Pol I was not dispersed and still localised at the single nucleolus and ESB (Fig. 5G-I), with an average separation of 76±39 nm between the ESB1 focus and the ESB. In bloodstream forms ESB1 overexpression therefore does not alter ESB number or form.

RNAseq transcriptome profiling of the ESB1 overexpression cell line showed a dramatic increase in mRNA levels, up to ~100× for VSGs and ESAGs transcribed from inactive BESs, while the mRNA level of VSG and ESAGs transcribed from the active BES remained unchanged (Fig. 5D, Extended Data Fig. 6A and confirmed using qRTPCR in Fig. 5F) arising from a ~10× increase in ESB1 mRNA (Fig. 5E). mRNAs transcribed from the specialised ESs containing metacyclic VSGs (MESs), normally expressed in the metacyclic life stage that is pre-adapted for transmission to the mammalian host, were similarly upregulated (Fig. 5D). Nascent inactive BES ESAG and VSG transcripts also dramatically increased (Extended Data Fig. 6). mRNA transcribed from all inactive BESs increased (Extended Data Fig. 6C) with promoter-proximal ESAGs tending to be more strongly affected than promoter-distal ESAGs and VSGs (Extended Data Fig. 6D), unlike the phenotype of VEX1 knockdown (Extended Data Fig. 6E). ESB1 overexpression is therefore sufficient to cause activation of inactive BES transcription although it may not be fully processive. All cells still expressed VSG221 (Extended Data Fig. 6F,G) therefore likely expressing multiple VSGs rather than switching to an alternative ES and VSG, whilst expression of procyclic form-specific surface proteins (procyclins) remained low (Extended Data Fig. 6A).

**Fig. 6:**
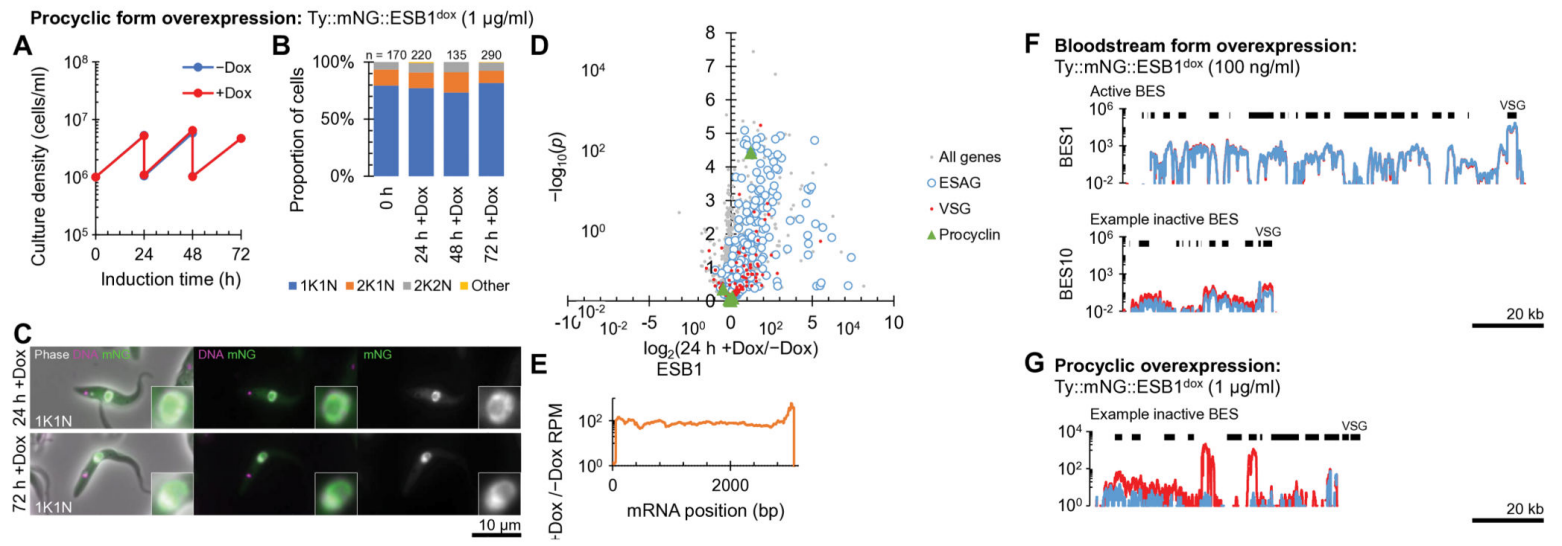
ESB1 overexpression in PCFs activates BES transcription without forming an ESB **A-E)** Cellular phenotype of mNG-tagged ESB1 overexpression in procyclic forms induced with 1 μg/ml doxycycline. **A)** Culture growth (with subculture) and **B)** counts of morphologically abnormal (‘Other’) after induction. *n* indicates number of cells counted from 1 induction, no changes *p* ≤ 0.05 from χ^2^ test. **C)** Example fluorescence images from *n* = 1 induction of overexpressed mNG-tagged ESB1 in procyclic forms. **D)** Volcano plot of change in mRNA abundance, *n* = 4 inductions (further time points in Extended Data Fig. 6H). *p* from two-tailed t test. **E)** Change in ESB1 ORF read coverage determined by RNAseq 24 h after induction, mean of *n* = 4 inductions. **F,G)** BES read coverage, determined by RNAseq 24 h after ESB1 overexpression, of **F)** the active and an example inactive BES in bloodstream forms (from Fig. 5) and **G)** an example inactive BES in procyclic forms, mean of *n* = 4 inductions. Extended in Extended Data Fig. 8.

Finally, we forced expression of tagged ESB1 in procyclic form cells (Fig. 6A-D, Extended Data Fig. 6H). Significant expression produced no growth or cytokinesis defects (Fig. 6A,B) and tagged ESB1 was nuclear but did not localise to a single extranucleolar ESB-like focus (Fig. 6C). RNAseq analysis showed a large increase (up to ~200×) in mRNA level for ESAGs, consistent with ESB1 activating transcription initiation at BES promoters which are normally inactive in the procyclic form (Fig. 6D). In this particular strain, we interrogated expression of the ESAGs and VSG from the sequenced BES^37^. Every ESAG transcribed from this BES was upregulated, typically ~3-5× and up to ~80× (Fig. 6F,G). In contrast, VSG mRNAs (both published and from our *de novo* assembly of the transcriptome) were not strongly upregulated (Fig. 6D). We did not see transcript from VSG 10.1, found in the sequenced BES, nor upregulation of any of the VSGs commonly expressed by this strain in bloodstream forms during mouse infection^38^. This is despite ~50× overexpression of ESB1 transcript relative to endogenous bloodstream form expression (Fig. 5E, Fig. 6E). As for tagged ESB1 overexpression in the bloodstream form, procyclin mRNA levels also remained unchanged (Fig. 6D). Hence ESB1 expression in procyclic forms activates BES transcription without forming an ESB; however, transcription is either not fully processive to the most distal gene (VSG) or there is additional machinery required for VSG transcript maturation, processing and/or stability not expressed in the procyclic form, e.g. CFB2^39^.

## Discussion

Antigenic variation in *T. brucei* relies on monoallelic expression of the VSG gene in the active BES. Our results provide the basis for a model whereby strong transcription activation of the active BES is counterbalanced by a strong repression of all other BESs, and has provided insights into ESB subdomains which orchestrate these different functions.

**Extended Data Fig. 7:**
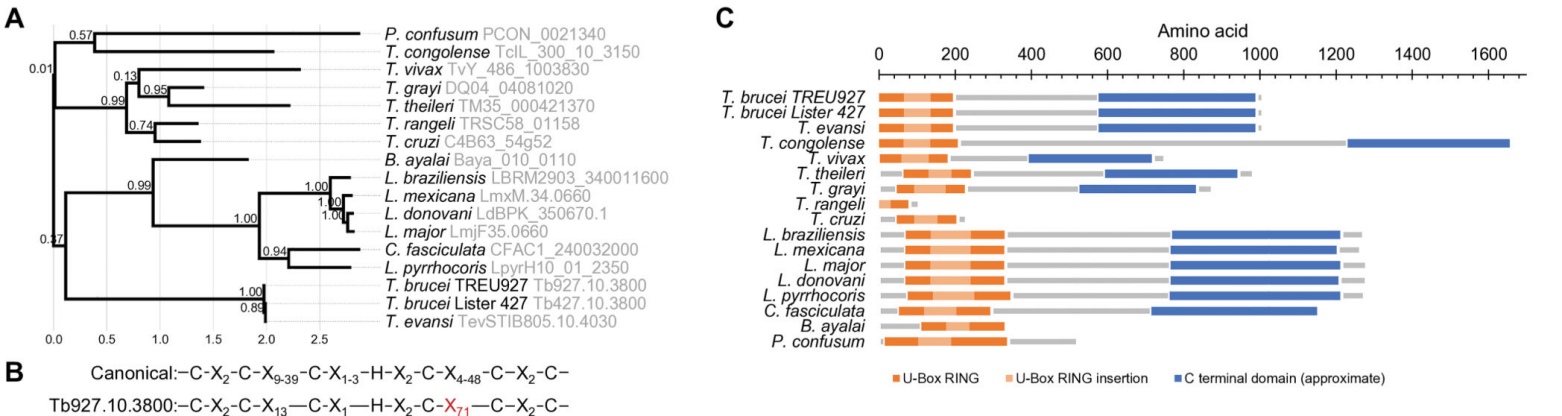
ESB orthologs among kinetoplastid parasites. **A)** Fast approximately maximum-likelihood phylogenetic tree of ESB1 and its orthologs. Node values are SH-like support. **B)** ESB1 U-box RING finger domain compared to the canonical sequence ^84^ showing a large insertion. **C)** Domain structure of ESB1 and its orthologs. From sequence alone, the C terminal domain could not be detected outside of trypanosomatids.

We have identified ESB1 as an ESB-specific protein and as an ES transcription activator enriched near the Pol I promoter. We show that ESB1 is necessary for the high level of transcription from the active BES and its overexpression activates only VSG-containing ESs and not procyclin loci. Importantly, both BESs and MESs are upregulated, and previous transcriptomics showed metacyclic forms have upregulated ESB1^40^, indicating VSG expression in the earliest VSG-expressing life cycle stage is ESB1-dependent. Ectopic expression of ESB1 in procyclic forms which never naturally express VSG was sufficient to activate BES promoter transcription, upregulating ESAGs located within a BES. However, ESB1 alone in procyclic forms was not sufficient for fully processive BES transcription and/or VSG mRNA processing. Interestingly, all trypanosomatid parasites, most of which do not undergo similar antigenic variation, have divergent ESB1 orthologs (Extended Data Fig. 7A). All orthologs have an N-terminal RING U-box domain, while the weakly conserved C terminal domain is not present in *Trypanosoma cruzi* and related *Trypanosoma* and, when it is present, has very low sequence similarity to *T. brucei* (Extended Data Fig. 7B,C). This raises the prediction that Pol I transcription of protein-coding genes and their regulation may occur in other trypanosomatid parasites.

ESB1 alone was also not sufficient to support formation of the Pol I and ESB1 focus, as overexpression of ESB1 did not give rise to an ESB-like body/bodies in the procyclic form or supernumerary ESB-like bodies in the bloodstream form. Moreover, multiple active BESs in multiple ESBs is not a stable state: in cells forced to express two VSGs from two BESs, both were recruited to a single ESB^41^. Given this and our ESB1 overexpression results suggests that the reasons for ESB absence (procyclic forms) or singularity (bloodstream forms) are likely to be more complex than a threshold level of ESB1 protein. Phase separation, common in nuclear compartment formation, amongst possible mechanisms for ESB formation where singularity could be achieved by emergent properties (Otswald ripening); however, ESB1 appears strongly chromatin-associated, perhaps acting as a single nucleation site. These are open hypotheses for future work, which may also have important implications for understanding switching between BESs.

Our work, taken with that of others, shows that the ESB is a complex nuclear body with multiple subdomains. The defining subdomain is a focus of Pol I around the active BES^7^, which also contains basal Pol I transcription factors^42^ and ESB1. This is associated with a highly SUMOylated focus (HSF)^21^. ESB1 is required for assembly of this subdomain. The BES is found in close proximity to one of the spliced leader array alleles^25^. Pol II transcription of this array generates spliced leader RNA necessary for processing of all transcripts into mRNA. Each spliced leader array allele is found in a Pol II transcription focus^27^ and the proximity of one allelic copy to the ESB BES/Pol I subdomain provides a mechanism for efficient processing of the large amount of VSG mRNA. BES association with the ESB spliced leader array/Pol II subdomain requires VEX2^25^ and the ESB BES/Pol I subdomain overlaps or is adjacent to one VEX1 and VEX2 nuclear focus^23–25^. We show that assembly of these foci are separable, with assembly of the VEX foci not dependent on ESB1 and vice-versa. Importantly we show that the Pol I and ESB1 focus is strictly singular. This enhanced appreciation of the ESB in terms of spatially defined subdomains raises the possibility that this reflects an intrinsic functional architecture.

## Methods

### Parasite strains and cell culture

*Trypanosoma brucei* Lister 427 bloodstream form (BSF) was used as its expression sites are sequenced^43^ and assembled into contigs^8^. BSFs were grown in HMI-9^44^ at 37°C with 5% CO_2_, maintained under ~2×10^6^ cells/ml by regular subculture. The active BES was BES1 containing VSG221 (also called VSG 427-2). *T. brucei* TREU927 procyclic form (PCF), selected as it is the original genome strain with genome-wide PCF localisation data^28,45^, were grown in SDM-79^46^ at 28°C, maintained between 6×10^5^ and 2×10^7^ cells/ml by regular subculture. We used PCF and BSF cell lines which express T7 RNA polymerase, Tet repressor, Cas9 and PURO drug selectable marker. These cell lines were generated using pJ1339, an expression construct which integrates into the tubulin locus^47^. To generate the Lister 427 BSF 1339 cell line, pJ1339 was linearised with HindIII and transfected into BSFs.

### Electroporation and drug selection

1-5 μg of linearised plasmid DNA or DNA from the necessary PCRs was purified by either phenol chloroform extraction (localisation screen) or ethanol precipitation (other experiments) then mixed with 3×10^7^ cells (BSFs) or 1×10^7^ cells (PCFs) in 100 μl of Tb-BSF buffer^48^. Transfection used the Amaxa Nucleofector IIb electroporator (program X-001, Lonza) in 2 mm gap cuvettes. Cells were transferred to 10 ml appropriate pre-warmed media for 6 h, then the necessary drugs added to select for successful construct integration. Clonal cell lines were generated (except for the localisation screen) by limiting dilution cloning. Cultures were maintained with drug selection for any genetic modifications, using 0.2 μg/ml (BSF) or 1.0 μg/ml (PCF) Puromycin Dihydrochloride, 5.0 μg/ml (BSF) or 10 μg/ml (PCF) Blasticidin S Hydrochloride, 2.0 μg/ml (BSF) or 15 μg/ml (PCF) G-418 Disulfate, 5 μg/ml (BSF) or 25 μg/ml (PCF) Hygromycin B, 2.5 μg/ml (BSF) or 5.0 μg/ml Phleomycin. Drug selection was removed for at least one subculture prior to an experiment.

### Medium-throughput BSF localisation screen for ESB proteins

Tagging candidates were selected using published mRNA abundance data (RNAseq)^49^, taking those with significantly upregulated transcripts (*p* < 0.05, two-tailed t-test) in BSFs relative to PCFs and prioritising those > 2.5× upregulated (Fig. 1B). Genes with unknown function were prioritised, and VSG genes and pseudogenes, ESAGs, genes related to ESAGs and known invariant surface glycoproteins were excluded. Some known proteins e.g. ISG65 and GPI-PLC were tagged as controls. We used other transcriptomic and ribosome footprinting datasets for further manual prioritisation^49–52,52,53^. Tagging was at the N terminus unless the protein had a predicted signal peptide, then the C terminus was tagged. We attempted tagging of 207 proteins and successfully generated 153 tagged cell lines, 7 had a nuclear signal (Extended Data Fig. 1).

### Endogenous tagging

To tag genes at the endogenous gene loci, we used long primer PCR and the pPOT plasmid series as the template to generate tagging constructs and, for BSF tagging, PCR to generate DNA encoding sgRNA with a T7 promoter^54,55^. mNeonGreen (mNG)^56^ was used for green fluorescent protein tagging, except for cell lines for ChIP where eYFP was used. pPOTv7-blast-mNG was used for the medium-throughput bloodstream form localisation screen. pPOTv6-blast-3Ty::mNG::3Ty was used for other experiments and for simplicity we refer to this as a 6×Ty::mNG tag. pPOTv7-hyg-tdTomato was used for tagging with a red fluorescent protein. PCR confirmed the correct fusion of the mNG CDS to the ESB1 CDS in PCFs.

### Exogenous expression and conditional knockout

For exogenous ESB1 (over)expression, the Tb927.10.3800 ORF was amplified by PCR from TREU927 genomic DNA (gDNA) and cloned into pDex577^57^ and pDex777^58^ with a 1×Ty::mNG combined fluorescence reporter and epitope tag. These are doxycycline-inducible constructs using a T7 promoter which integrate into the transcriptionally silent minichromosome repeats. pDex577/pDex777 constructs were linearised with NotI before transfection.

We titrated doxycycline concentrations to achieve desirable exogenous Ty::mNG::ESB1 expression level by comparison to a cell line expressing 6×Ty::mNG::ESB1 from the endogenous locus, using light microscopy, Western blot (Extended Data Fig. 3A,B) and RNAseq. We selected conditions to give (i) approximately endogenous expression level in BSFs (pDex577 with 10 ng/ml doxycycline), (ii) overexpression sufficient to generate an aberrant BSF phenotype (pDex577 with 100 ng/ml doxycycline, Extended Data Fig. 3B) or (iii) high overexpression in PCFs (pDex777 with 1 μg/ml doxycycline, Fig. 6E).

RNAseq confirmed no major perturbation of cellular transcripts in BSFs expressing exogenous Ty::mNG::ESB1 from pDex577 with 10 ng/ml doxycycline (Extended Data Fig. 3D). We then deleted both endogenous ESB1 alleles (Extended Data Fig. 3C) while maintaining the cell line with 10 ng/ml doxycycline to generate the cKO cell line. For gene knockout, we used long primer PCR to generate deletion and sgRNA constructs^54,55^, using pPOTv7 Hyg and pPOTv6 Blast. We confirmed knockout by PCR from genomic DNA, testing for loss of the target gene CDS and replacement of the target gene CDS with the drug selection marker. The cKO phenotype was observed by washing out the doxycycline.

### Endogenous locus ORF modification/loss PCR validation

Key endogenous locus modifications were validated by PCR using template gDNA extracted using the DNeasy Blood & Tissue Kit (Qiagen). Primer pairs (Extended Data Table 2) spanned from the endogenous DNA sequence to the integrated DNA: For deletions, the gene 5′ UTR to the drug selection marker ORF (Extended Data Fig. 2G, Extended Data Fig. 3C), and for tagging, the gene ORF to the fluorescent tag ORF (Extended Data Fig. 2B,G, Extended Data Fig. 3C). PCR product size was checked by agarose gel electrophoresis (for primer sequences see **Error! Reference source not found.**). In cases where both gene alleles were modified the first allele modification was confirmed by PCR, before the second allele was modified and confirmed.

### Inducible RNAi knockdown

For inducible ESB1, VEX1 or VEX2 RNAi knockdown, we cloned a fragment (primer sequences in Extended Data Table 3) of the target gene ORF into a new doxycycline-inducible RNAi construct, pDRv0.5 (Supporting Information). This gives two copies of the fragment in reverse complement separated by a 150 nt stuffer. Two opposing T7 promoters under the control of doxycycline drive transcription of the resulting “stem-loop”. Cells were transfected with NotI linearised plasmid and selected using Hygromycin B. The construct integrates into the rRNA array. RNAi was induced using 1 μg/mL doxycycline.

To confirm effective knockdown, we introduced RNAi constructs into cell lines expressing an endogenously tagged copy of the target protein whose knockdown was confirmed by light microscopy (Extended Data Fig. 5C, Fig. 4L) and/or Western blot (Extended Data Fig. 5D) and/or RNAseq to determine transcript abundance of the target gene (Fig. 4J).

For ESB1 RNAi knockdown in cell lines expressing endogenously tagged RPA2, SUMO, VEX1 or VEX2, we confirmed ESB1 knockdown by checking for the expected growth rate defect and change in proportion of cells at different cell cycle stages.

### Western blotting

Expression of endogenously tagged and exogenously (over)expressed proteins was confirmed by Western blotting, using 1:100 anti-mNG (mouse monoclonal IgG2c, ChromoTek 32f6, RRID: AB_2827566) or 1:100 anti-TY (from BB2 hybridoma, mouse monoclonal IgG1^59^) primary antibody and anti-mouse HRP-conjugated secondary antibody.

### Induction time series

RNAi and cKO cell lines were analysed as induction time series with paired induced and uninduced samples. Cells were subcultured to 1×10^5^ cell/ml (BSFs) or 1×10^6^ cells/ml (PCFs), one sample without and one with the appropriate doxycycline concentration for induction. Each 24 h, the culture density was measured, samples taken, then the remaining cells subcultured to 1×10^5^ cell/ml (BSFs) or 1×10^6^ cells/ml (PCFs), including doxycycline in the induced sample. For cultures with a strong growth defect, the culture was centrifuged at 1200 g for 5 min, the cell pellet resuspended in fresh medium, and doxycycline added if needed, to maintain constant conditions. Growth defects were tested with two-tailed t-tests on log-transformed cumulative growth.

### Microscopy

Unless otherwise noted, light microscopy was carried out on live cells adhered to glass with DNA stained with Hoechst 33342^60^, captured on a DM5500 B (Leica) widefield epifluorescence microscope using a plan apo NA/1.4 63× phase contrast oil-immersion objective (Leica, 15506351) and a Neo v5.5 (Andor) sCMOS camera using Micro-Manager (version 1.4)^61^.

Kinetoplasts (K, mitochondrial DNA) and nuclei (N) in cells were counted from micrographs as a measure of cell cycle stage. K division normally precedes N division, giving 1K1N, 2K1N then 2K2N cells prior to cytokinesis. Cells with abnormal K/N numbers were classified as ‘other’. Change in cell cycle stage distribution was tested with the χ^2^ test.

Spacing of point-like structures, one in green and one in red, was carried out by fitting a Gaussian in each channel then calculating centre point separation using ImageJ (version 1.50)^62,63^. Prior to analysis, chromatic aberration was corrected using reference images of 0.1 μm TetraSpeck multi-colour fluorescent beads (ThermoFisher) adhered to glass^64^ and measurement error determined using green-red spacing in independent chromatic aberration-corrected images of the multi-colour fluorescent beads.

For blinded counts, one researcher identified and cropped in-focus nuclei of 1K1N cells from a mixture of test and control samples and saved each image with a randomised file name while generating an index. A second researcher classified the nuclei, then unblinded using the index file.

For anti-VSG221 immunofluorescence, slides were prepared as for live cell microscopy then the cells fixed with 2% formaldehyde for 5 min. Slide were then washed three times with PBS, incubated with 1:2000 polyclonal rabbit anti-VSG^65^ for 1 h, washed three times with PBS, incubated anti-rabbit Alexa Fluor 647-conjugated secondary antibody for 1 h, washed three times with PBS and mounted with 50 mM phosphate-buffered 90% glycerol^60^.

### Transcriptomic analysis

RNA samples for each experiment were purified at the same time by inducing separate samples at appropriately staggered intervals. A paired uninduced sample, maintained by the same pattern of subculture, was generated for each induction time point. From this time series, a time of primary interest was identified and three further paired samples were prepared. For each, 10^8^ cells were harvested by centrifugation at 3200 g for 90 s, the supernatant discarded, and the pellet resuspended in 1 ml serum free HMI-9. The suspension was centrifuged again at 10000 g for 30 s, the supernatant discarded by pipetting and the pellet flash frozen in a −78°C dry ice/ethanol bath. Total processing time is under 4 min. RNA was extracted using the RNeasy Mini Kit (Qiagen), eluted in 30 μl nuclease-free water and stored at −80°C. For RNAseq, mRNA was enriched by polyA selection, cDNA generated by reverse transcription using a poly-dT primer then subjected to 100 bp paired end sequencing (BGISEQ-500) with a nominal insert size of 200 bp and >70,000,000 reads per sample.

To quantify transcript abundance from whole mRNAs rather than CDSs, we first mapped the 5′ and 3′ UTRs using all our BSF Lister 427 RNAseq data. Spliced leader acceptor sites (SLASs) and polyadenylation sites (PASs) were identified and assigned to protein-coding genes in the TriTrypDB^66,67^ release 45 of the *T. brucei* Lister 427 2018 genome using SLaPMapper^68^. SLASs and PASs only observed once, used for <5% of transcripts from a gene or within a CDS were excluded – no attempt was made to correct CDSs based on SLASs/PASs. The most distant SLAS and PAS, within 5 kb, of the CDS defined the 5’ and 3’ UTR respectively.

To quantify transcript abundance, fastq reads were mapped to the appropriate transcriptome using BWA-MEM (version 0.7.17) with default settings. Our Lister 427 2018 transcripts were used for 427 BSF samples and TriTrypDB^66,67^ release 45 for *T. brucei* TREU927 annotated transcripts for 927 PCF samples. An additional contig for the single sequenced and assembled *T. brucei* TREU927 BES^37^ was generated from NCBI GenBank AC087700 (BES) and AF335471 (VSG), from which the ESAG and VSG ORFs were identified and appended to the TREU927 transcriptome.

ESAGs have very similar sequences, therefore transcript abundances were determined using uniquely mapped reads. Alignments were filtered to include only correctly mapped pairs with MAPQ>10, excluding unmapped reads, secondary alignments and PCR or optical duplicates, using samtools view (version 1.7) with flags -q 10, -F 0x504 and -f 0x02. We confirmed that this accurately maps reads to the correct BES using simulated reads^69^. Using ART (version 2016-06-05)^70^, we generated an error model (using all RNAseq data). Using this model and the measured insert size of 208±78 bp, we simulated data with 500-fold coverage of Lister 427 2018 transcripts, and aligned them to the Lister 427 genome. Without filtering, between 54.9% (BES5) to 90.2% (BES10) of simulated reads were mapped to the correct BES. With filtering, this improved to >99.75% for all BESs.

Reads per kilobase per million reads (RPKM) was calculated from samtools idxstats. Mean read coverage was calculated from samtools depth with flags -aa -d 10000000, then converted to counts per million reads (CPM). For time points with a single replicate, z intervals were calculated from variation between uninduced samples (n = 3). Standard deviation of log fold change for transcripts binned by RPKM (20 bins, *n* > 70 genes per bin) was calculated and fitted to a third order polynomial for plotting. For time points with multiple replicates, mean and two-tailed t-test p value of log_2_ fold change were calculated for volcano plots.

Immature/nascent transcripts were quantified by filtering the alignments for reads spanning a SLAS or PAS, indicating trans splicing or polyadenylation respectively may have not yet occurred. Reads were scored by the sum frequency of use of the spanned sites (1 if the only site, 0.05 if spanning a site used 5% of the time, 0.97 if spanning two sites used 63% and 34% of the time respectively, etc.) then normalised to score per 1,000,000 reads (ie. RPM-like).

Active BES VSG (VSG221) quantitative reverse transcription PCR (qRT-PCR) used a one-step protocol from total RNA, with β-tubulin as a control (primer sequences in Extended Data Table 4). Total RNA was diluted to 500 ng/μl based on OD260, and qRT-PCR performed using the QuantiTect SYBR Green RT-PCR Kit (Qiagen, 204243) using the manufacturer-recommended reaction composition and thermocycle on a Mx3000P QPCR machine (Agilent). Specific PCR product was confirmed by gel electrophoresis, product melt curve analysis and no template and no primer controls. A six step three-fold dilution series from 1:3^0^ (1:1) to 1:3^6^ (1:279) of parental cell line RNA was used to confirm VSG and tubulin critical cycles fell in the linear range. Mean VSG221 to tubulin critical cycle was determined in triplicate using 1:10 diluted RNA samples and MxPro QPCR Software (Agilent).

For de novo transcriptome assembly we used Trinity (version 2.11.0) guided by Harvard FAS best practices^71^. Sequencing errors were first corrected using Rcorrector (version 1.0.4)^72^ and uncorrectable reads were removed, then any remaining adapters and low quality sections trimmed with Trim Galore! (version 0.6.0) with flags --length 36, -q 5, --stringency 1 and -e 0.1. Finally, read ends which exactly matched 4 or more bases of the 3′ end of the *T. brucei* spliced leader sequence were trimmed. Trinity using default settings generated the assembly.

### ChIPseq

For ChIPseq we used the following optimised protocol^73^. 5x10^8^ BSF expressing YFP::ESB1 at 1x106 cells/ml were fixed with 1/8 volume of formaldehyde (50 mM HEPES-KOH pH 7.5, 100 mM NaCl, 1 mM EDTA, 0.5 mM EGTA and 8% formaldehyde) for 20 min at room temperature, followed by addition of 1/13 volume of 2 M glycine and kept on ice. The fixed cells were rinsed with 35 ml PBS, resuspended in 35 ml Lysis Buffer 1 (50 mM HEPES-KOH pH 7.5, 140 mM NaCl, 1 mM EDTA, 10% glycerol, 0.5% NP-40, 0.25% Triton X-100, protease inhibitors), and centrifuged at 4000 g for 15 min. The pellet was resuspended in 35 ml Lysis Buffer 2 (10 mM Tris-HCl pH 8.0, 200 mM NaCl, 1 mM EDTA, 0.5 mM EGTA, protease inhibitors), and centrifuged at 4000 g for 15 min. The pellet was resuspended in 4 ml Lysis Buffer 3 (10 mM Tris-HCl pH 8.0, 100 mM NaCl, 1 mM EDTA, 0.5 mM EGTA, 0.1% Na-Deoxycholate, 0.5% N-lauroylsarcosine, protease inhibitors) and sonicated (27 s on/30 s off, 8 cycles) using a VCX 130 PB (Sonics & Materials Inc.). 1/10 volume of 10% Triton X-100 was added to the sonicated lysate and centrifuged at 21000 g for 10 min to pellet debris and the supernatant was collected. YFP-tagged proteins were immunoprecipitated with rabbit anti-GFP (Invitrogen, A11122, RRID: AB_221569) that were pre-conjugated with Protein-A magnetic beads (Dynal). Beads were washed with 1 ml RIPA buffer (50 mM HEPES-KOH pH 7.5, 500 mM LiCl, 1 mM EDTA, 1.0% NP-40, 0.7% Na-Deoxycholate) seven times and rinsed with TE supplemented with 50 mM NaCl. DNA was eluted with 200 μl elution buffer (50 mM Tris-HCl pH 8.0, 10 mM EDTA, 1.0% SDS) at 65°C for 30 min. Crosslinking was reversed by incubating at 65°C overnight. The sample was treated with RNaseA (0.4 mg/ml, QIAGEN) at 37°C for 2 hr and Proteinase K (0.420 mg/ml, Life technologies Ltd) at 55°C for 2 hr the was purified using a PCR purification kit (QIAGEN).

Both input and ChIP DNA were sequenced by 50 bp single-end sequencing (DNBSEQ). ChIP/input ratio was calculated from reads uniquely mapped to the 2018 resequence of T. brucei Lister 427, using BWA and samtools. Only reads with MAPQ > 3 were included and unmapped reads, secondary alignments and read PCR or optical duplicates were excluded, using samtools with flags -q 3 and -F 0x504. Mean ChIP/input ratio was calculated for each BES contig and calculated genome-wide in 2 kb bins. Bins with < 4 uniquely mapped input DNA reads were classed as unanalysable due to either insufficient input DNA or an insufficiently unique sequence for mapping of 50 bp reads.

## Extended Data

**Extended Data Fig. 8:**
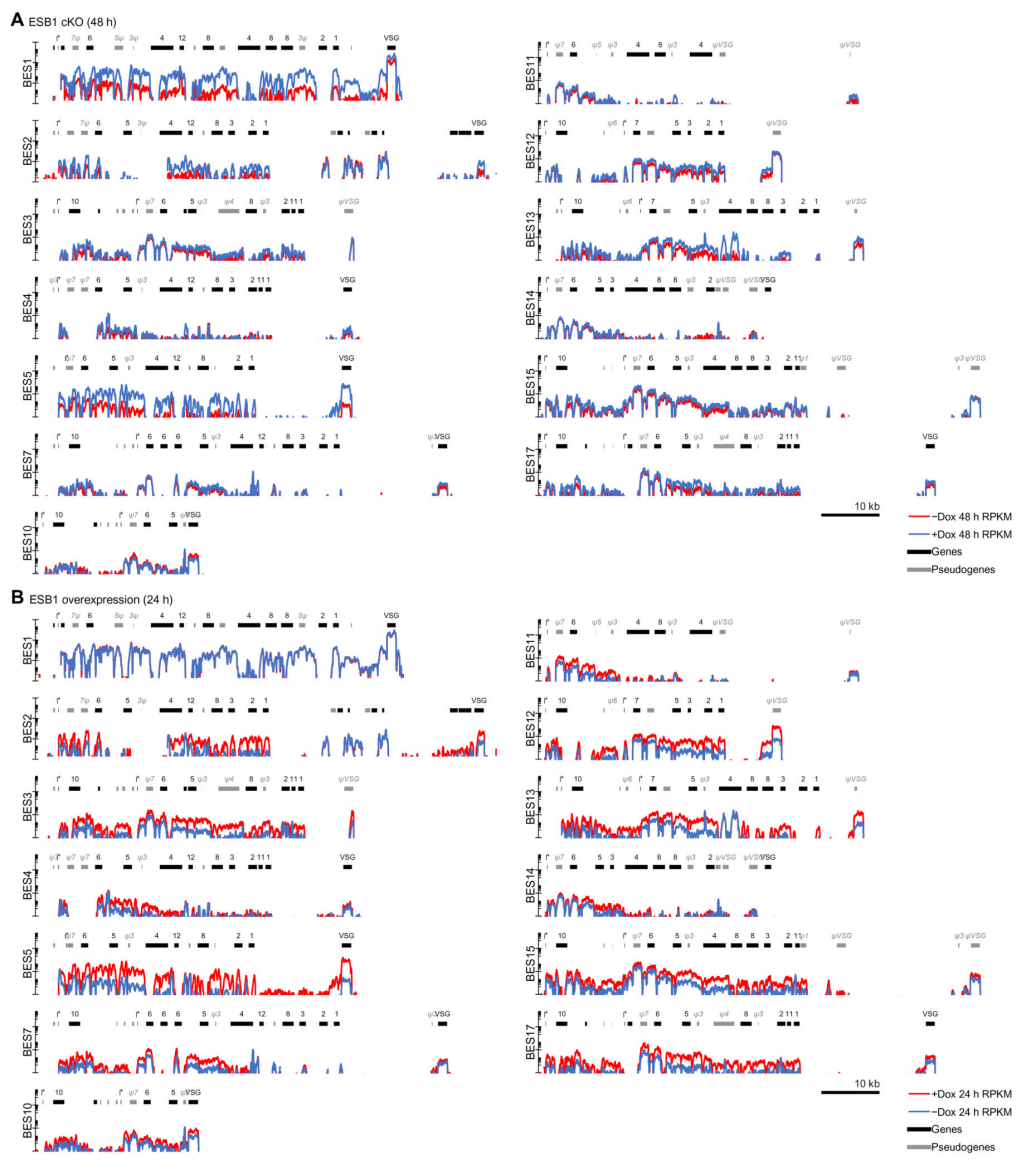
Extended presentation of RNAseq data showing the active and inactive BESs. **A,B)** Read coverage of the active BES (BES1) in comparison to all sequenced inactive BESs, determined by RNAseq, for **A)** the BSF ESB1 cKO (characterised in Fig. 2A-F) 48 h after induction and **B)** the BSF ESB1 overexpression (characterised in Fig. 5A-F) 12 h after induction. The latter is an extended version of Fig. 6F. Mean of *n* = 4 inductions.

## Supplementary Material

Extended Data Figure 2 source data

Extended Data Figure 5 source data

Extended Data Figure 6 source data

Figure 1 source data

Figure 2 source data

Figure 4 source data

Figure 5 source data

Figure 6 source data

Supplemental Data

Supplemental Tables S1-4

## Data Availability

RNAseq and ChIPseq data are available via the NCBI sequencing read archive (SRA) under BioProject accession number PRJNA784098. Further raw data are provided for Figures 1, 2, 4, 5, 6 and Extended Data Figures S2, S5, S6.
