# Supplementary material for: Stage-specific transcription activator ESB1 regulates monoallelic antigen expression in Trypanosoma brucei
Source: Nat Microbiol. 2022 Jul 25;7(8):1280–90. doi: 10.1038/s41564-022-01175-z (PMC9352583; doi:10.1038/s41564-022-01175-z)
Supplement: Supplementary file 1 [file 41564_2022_1175_MOESM1_ESM.pdf]

nature portfolio

https://doi.org/10.1038/s41564-022-01175-z

Supplementary information

Stage-specific transcription activator ESB1 regulates monoallelic antigen expression in *Trypanosoma brucei*

In the format provided by the authors and unedited

Extended Data Table 1. Previously described proteins involved in VSG expression or monoallelic exclusion.

Group	Protein	Gene ID	Reference
ESB-associated VSG exclusion	VEX1	Tb927.11.16920	23
	VEX2	Tb927.11.13380	24
ESB-associated transcription activators	SUMO	Tb927.5.3210	21
	SIZ1	Tb927.9.11070	21
	SNF2PH	Tb927.3.2140	22
Pol I basal transcription factors	CITFA-7	Tb927.7.2600	42,74
Cohesin	SCC1	Tb927.7.6900	29
Telomere-associated	ORC1	Tb927.11.7216	9
	RAP1	Tb927.11.370	12
	TIF2	Tb927.3.1560	10,75
	TRF	Tb927.10.12850	10,76
	JBP1	Tb927.11.13640	11
	JBP2	Tb927.7.4650	11
Specialised chromatin & remodelling	DOT1B	Tb927.1.570	15
	CAF-1B	Tb927.10.7050	13
	ASF1	Tb927.1.630	13
	ISWI	Tb927.2.1810	16
	NLP	Tb927.2.1810	17
	BDF2	Tb927.10.7420	18
	BDF3	Tb927.11.10070	18
	POB3	Tb927.10.14390	14
	SPT16	Tb927.3.5620	14
	DAC3	Tb927.2.2190	19
	SAP	Tb927.10.4440	77
Nuclear envelope	NUP-1	Tb927.2.4230	78
	NUP-2	Tb927.9.6460	79
Specialised chromatin	TDP1	Tb927.3.3490	20,80
	H3.V	Tb927.10.15350	8
	H4.V	Tb927.2.2670	8
Chromatin	H1	Tb927.11.1790, Tb927.11.1800, Tb927.11.1860, Tb927.11.1870, Tb927.11.1880	65
	H3	Tb927.1.2430, Tb927.1.2450, Tb927.1.2470, Tb927.1.2490, Tb927.1.2510, Tb927.1.2530, Tb927.1.2550	13
DNA damage	ATR	Tb927.11.14680	81
Membrane	PIP5Pase	Tb927.11.6270	82,83
	PIP5K	Tb927.10.3890	82
	PLC	Tb927.11.5970	82

Extended Data Table 2. Primer sequences used for PCR validation of genetic modification.

Primer	Primer direction	Primer sequence
ESB1 5’ UTR	Forward	CTTTTGCAGTTGACATACCATGCTTTC
ESB1 ORF	Reverse	CGAAGTTTCTTGAACACGAG
HYG (hydromycin) ORF	Reverse	CAACGTGACACCCTGTGCTGCAC
BSR (blasticidin) ORF	Reverse	GCTTTGATCCCAGGATGCAGATC
NEO (neomycin) ORF	Reverse	CTCGTCAAGAAGGCGATAGAAG
mNG ORF	Forward	GTCTCGAAAGGTGAGGAAGACAATATG

Extended Data Table 3. Primer sequences used for generation of RNAi constructs.

Gene	Gene ID	Forward primer sequence	Reverse primer sequence
ESB1	Tb927.10.3800	tatGGTCTCaCTCTcaccaactgtgcgtgaagtg	aaaGGTCTCtCACAaggcaagtcttccttccgtg
VEX1	Tb927.11.16920	tatGGTCTCaCTCTccacacctaaccaactg	aaaGGTCTCtCACAacttacagccgttgttg
VEX2	Tb927.11.13380	tatGGTCTCaCTCTgccgccagatatcaccatca	aaaGGTCTCtCACAcgaaatcggcgtacgtgttc

Extended Data Table 4. Primer sequences used for qRT-PCR.

Gene	Gene ID	Forward primer sequence	Reverse primer sequence
VSG221	Tb427_000016000	TTTCTGCAGCGGTCACTATG	GCCTGTTTTGCCAGCTATTC
β-tubulin	Tb427_010017100	ATGAGCAGATGCTGAACGTG	GCCAGTGTACCAGTGCAAGA
